# Impact of Side Chains in 1‐*n*‐Alkylimidazolium Ionomers on Cu‐Catalyzed Electrochemical CO_2_ Reduction

**DOI:** 10.1002/advs.202406281

**Published:** 2024-10-31

**Authors:** Young In Song, Bohak Yoon, Chanwoo Lee, Dogyeong Kim, Man Ho Han, Hyungu Han, Woong Hee Lee, Da Hye Won, Jung Kyu Kim, Hyo Sang Jeon, Jai Hyun Koh

**Affiliations:** ^1^ Clean Energy Research Center Korea Institute of Science and Technology (KIST) Seoul 02792 Republic of Korea; ^2^ School of Chemical Engineering Sungkyunkwan University (SKKU) Suwon 16419 Republic of Korea; ^3^ Department of Chemistry Chicago Center for Theoretical Chemistry Institute for Biophysical Dynamics, and James Franck Institute The University of Chicago Chicago IL 60637 USA; ^4^ Division of Energy & Environment Technology KIST School University of Science and Technology Seoul 02792 Republic of Korea; ^5^ Department of Chemical and Biological Engineering Korea University Seoul 02841 Republic of Korea; ^6^ KHU‐KIST Department of Converging Science and Technology Kyung Hee University Seoul 02477 Republic of Korea; ^7^ Technological Convergence Center Korea Institute of Science and Technology (KIST) Seoul 02792 Republic of Korea

**Keywords:** binder, Cu catalyst, DFT, ionomer, CO_2_ reduction

## Abstract

This study presents the impact of the side chains in 1‐*n*‐alkylimidazolium ionomers with varying side chain lengths (C_n_H_2n+1_ where *n* = 1, 4, 10, 16) on Cu‐catalyzed electrochemical CO_2_ reduction reaction (CO_2_RR). Longer side chains suppress the H_2_ and CH_4_ formation, with the *n*‐hexadecyl ionomer (*n* = 16) showing the greatest reduction in kinetics by up to 56.5% and 60.0%, respectively. On the other hand, C_2_H_4_ production demonstrates optimal Faradaic efficiency with the *n*‐decyl ionomer (*n* = 10), a substantial increase of 59.9% compared to its methyl analog (*n* = 1). Through a combination of density functional theory calculations and material characterization, it is revealed that the engineering of the side chains effectively modulates the thermodynamic stability of key intermediates, thus influencing the selectivity of both CO_2_RR and hydrogen evolution reaction. Moreover, ionomer engineering enables industrially relevant partial current density of –209.5 mA cm^−2^ and a Faradaic efficiency of 52.4% for C_2_H_4_ production at 3.95 V, even with a moderately active Cu catalyst, outperforming previous benchmarks and allowing for further improvement through catalyst engineering. This study underscores the critical role of ionomers in CO_2_RR, providing insights into their optimal design for sustainable chemical synthesis.

## Graphical abstract

This study demonstrates the impact of 1‐*n*‐alkylimidazolium ionomers with varying side chain lengths (C_n_H_2n+1_ where *n* = 1, 4, 10, 16) on Cu‐catalyzed electrochemical CO_2_ reduction. Longer side chains suppress H_2_ and CH_4_ formation, while C_2_H_4_ production shows optimal Faradaic efficiency with *n* = 10, highlighting the critical role of ionomer structure in CO_2_RR selectivity.

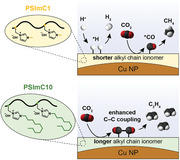

## 1 Introduction

Electrochemical CO_2_ reduction reaction (CO_2_RR) is a sustainable approach to convert CO_2_ and H_2_O into valuable fuels and chemicals using renewable electricity.^[^ ^1^, ^2^, ^3^ ^]^ Cu‐based catalysts have been the subject of significant research for CO_2_RR among different elements due to their unique capability of producing valuable hydrocarbons (e.g., CH_4_, C_2_H_4_) and alcohols.^[^ ^4^, ^5^ ^]^ However, achieving high activity and selectivity toward a specific product remains challenging due to the competing hydrogen evolution reaction (HER), high activation barriers in rate‐determining steps (RDS), etc.^[^ ^6^ ^]^ Ionomers have recently begun to be auditioned as binders for CO_2_RR to address these challenges.^[^ ^7^, ^8^, ^9^, ^10^, ^11^, ^12^, ^13^, ^14^, ^15^, ^16^, ^17^ ^]^ For example, Nafion, Sustainion XA‐9, and poly(terphenyl piperidinium) have shown distinct effects on Cu‐catalyzed CO_2_RR selectivity.^[^ ^9^ ^]^ These effects are attributed to the interplay between charge transfer through the ionomers and their hydrophobicity, suggesting that CO_2_RR selectivity is intricately linked to their chemical structures. Polyacrylic acid, fluorinated ethylene propylene, and Nafion have also demonstrated their critical roles in modulating catalytic performance by influencing the local concentration of H_2_O and CO_2_ near the catalyst surface.^[^ ^10^ ^]^ These pioneering investigations underscore the untapped potential of optimizing electrode surfaces with them to enhance both the activity and selectivity of CO_2_RR.

Most studies in the literature have focused on comparing the catalytic performance using a set of commercially available ionomers such as Nafion and Sustainion.^[^ ^7^, ^8^, ^9^, ^10^, ^11^, ^12^, ^13^ ^]^ However, these ionomers differ in their chemical structures, including polymer backbones, ion‐conducting groups, side chains, and other moieties. This focus has left a significant gap in understanding the relationship between the chemical structure of ionomers and CO_2_RR performance. Beyond the well‐recognized requirements for high ionic conductivity and robust chemical and mechanical stability,^[^ ^18^, ^19^ ^]^ the intricate mechanisms by which ionomers contribute to CO_2_RR need a more comprehensive understanding.^[^ ^20^ ^]^ It is thus important to conduct systematic research on ionomers by altering specific moieties of interest while maintaining the rest of the moieties constant. Such an approach will allow for a deeper understanding of the impact of these moieties on the CO_2_RR performance. This knowledge can then be utilized to develop tailored ionomers that enable more efficient and selective CO_2_RR.

Herein, we investigate the role of the side chains of 1‐*n*‐alkylimidazolium ionomers in Cu‐catalyzed CO_2_RR. These styrene‐based ionomers were systematically designed and synthesized to contain *n*‐alkyl side chains of varying lengths (C_n_H_2n+1_ where *n* = 1, 4, 10, 16) while sharing the identical polystyrene backbone and imidazolium ion‐conducting group. Electrochemical CO_2_RR was then conducted using these ionomers as binders to examine how the structural differences in the side chains impact the catalytic activity and selectivity. The production rates of both H_2_ and CH_4_ decline as side chain lengths increase, with the *n*‐hexadecyl ionomer (*n* = 16) demonstrating the most pronounced reduction. On the other hand, the C_2_H_4_ production shows an optimal Faradaic efficiency with the *n*‐decyl ionomer (*n* = 10), demonstrating the importance of optimizing side chain length in catalytic performance. By integrating density functional theory (DFT) calculations, hydrophobicity, and ionic conductivity assessments, we elucidate key intermediates in the kinetics of both CO_2_RR and HER and rationalize the origins of the selectivity.

## 2 Results and Discussion

**Figure 1:**
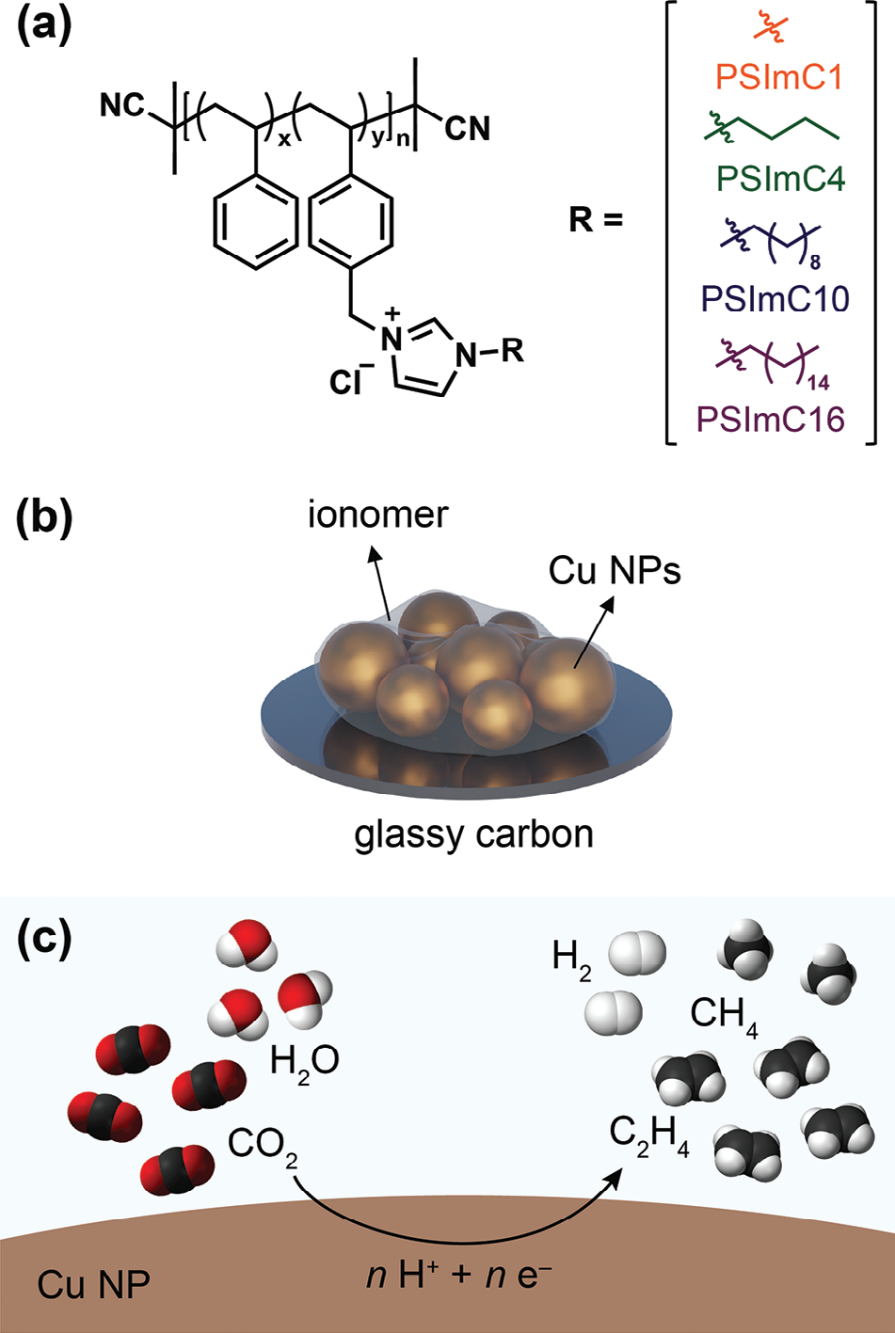
a) Chemical structures of 1‐*n*‐alkylimidazolium‐containing ionomers with different alkyl side‐chain lengths. b) Schematic representation of the glassy carbon electrode coated with the Cu nanoparticles (NPs) and 1‐*n*‐alkylimidazolium ionomers. c) Schematic illustration of electrochemical CO_2_ reduction process on the ionomer‐coated Cu NPs electrode.

A series of styrene‐based ionomers containing 1‐*n*‐alkylimidazolium groups with varying alkyl chains were synthesized via reversible addition‐fragmentation chain‐transfer (RAFT) polymerization, followed by functionalization reactions (Figure S1, Supporting Information). The RAFT polymerization yielded a well‐defined poly(styrene‐*co*‐4‐vinylbenzyl chloride) (P(S‐*co*‐VBC)) with a relatively low dispersity of 1.22, which serves well for studying structure‐property relationships (Figure S2, Supporting Information). Subsequently, functionalization reactions with a series of 1‐*n*‐alkylimidazoles were conducted using the P(S‐*co*‐VBC) as the parent copolymer to synthesize ionomers with 1‐*n*‐alkylimidazolium groups of different side chain lengths. The detailed synthetic procedures, gel permeation chromatography, ^1^H NMR spectra, FT‐IR spectra, thermal stability, and copolymerization and characterization data for these ionomers are provided in the Supporting Information (Experimental, Figures S2–S5 and Table S1). 1‐methylimidazole, 1‐*n*‐butylimidazole, 1‐*n*‐decylimidazole, and 1‐*n*‐hexadecylimidazole were used in this work. The corresponding ionomers synthesized using these compounds are denoted as PSImC1, PSImC4, PSImC10, and PSImC16, respectively (**Figure** **1**a).

Next, these ionomers were employed as binders for Cu catalysts on glassy carbon substrates for electrochemical CO_2_RR (Figure 1b,c). Commercial polycrystalline Cu nanoparticles (NPs) with an average size of 25 nm were selected as catalysts because of their modest catalytic performance on CO_2_RR. This selection was made to prioritize the examination of the effect of the ionomers on CO_2_RR performance rather than focusing on the intrinsic activity of the catalysts. The crystal structure of the Cu NPs was investigated by X‐ray diffraction, revealing (111), (200), and (220) facets (Figure S6, Supporting Information). For the preparation of electrodes, the Cu NPs and ionomers were mixed in methanol at a 1:1 mass ratio, and the resulting dispersion was sonicated for 30 min to form a catalyst ink. This ink was then cast onto a glassy carbon substrate to prepare an electrode (Figure S7, Supporting Information), with both catalysts and ionomer loaded at 0.1 mg cm^−2^. These electrodes were subjected to characterization prior to electrochemical experiments.

**Figure 2:**
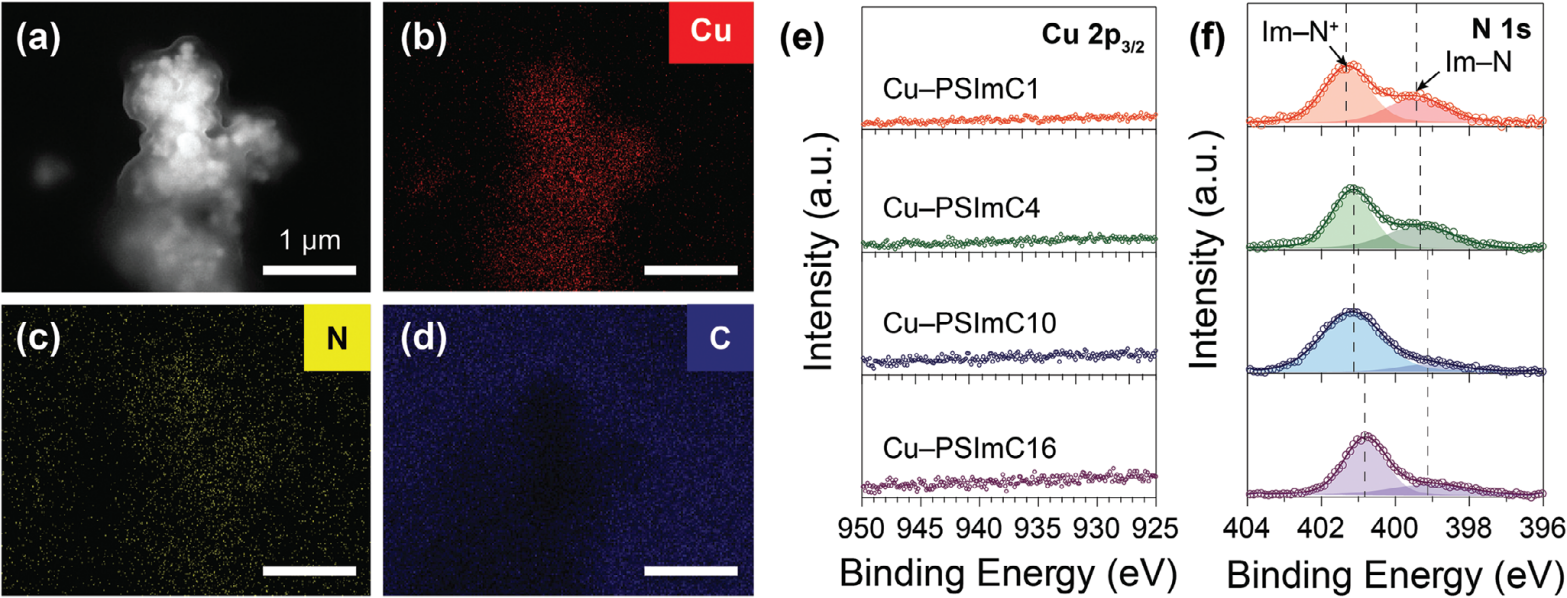
Characterization of the electrodes coated with the Cu NPs and as‐synthesized 1‐*n*‐alkylimidazolium ionomers. a) SEM image of Cu–PSImC10 and corresponding elemental mapping for b) Cu, c) N, and d) C (scale bar = 1 µm). e,f) X‐ray photoelectron spectroscopy of all electrodes in the (e) Cu 2*p*_3__/2_ and (f) N 1s regions.

**Figure** **2**a–d shows an SEM image of the representative Cu–PSImC10 sample and the corresponding elemental maps. The ionomer and Cu NPs are evenly distributed, as evidenced by the Cu and N maps (Figure 2b,c), which show the overlap of the imidazolium group of the ionomer with the distribution of Cu. Notably, the particles appear larger than the original size of around 25 nm, and semi‐transparent layers are present on top of all the prepared electrodes (Figure S8b–e, Supporting Information). On the other hand, the control sample of Cu NPs prepared without ionomer (Figure S8a, Supporting Information) exhibits the Cu NPs that retain their original particle size. This led to the hypothesis that these electrodes are composed of denser Cu NPs located lower in the coating, with less dense ionomer layers on top. To test this hypothesis, X‐ray photoelectron spectroscopy (XPS) was conducted on all electrodes and the control sample. No peaks are found in the region of Cu 2*p*_3__/2_ for all electrodes (Figure 2e), whereas distinct peaks corresponding to Cu^0^/Cu^+^ and Cu^2^^+^ are shown for the control sample (Figure S11b, Supporting Information). This implies that a layer of ionomers is coated on top of Cu NPs and is thick enough to hinder the detection of Cu signals by the surface‐sensitive XPS technique.^[^ ^21^ ^]^ In the N 1s regions (Figure 2f), the major peaks with higher binding energy (BE) ranging from 400.8 to 401.3 eV are assigned to the delocalized imidazolium N cations (Im−N^+^). Meanwhile, the minor peaks with lower BE from 399.1 to 399.5 eV are assigned to the non‐ionic N atoms (Im–N) in the 1‐*n*‐alkylimidazolium rings.^[^ ^22^ ^]^ The peak positions for both Im−N^+^ and Im–N shift toward lower BE values with the extension of the alkyl side chains. This shift occurs because longer and more electron‐donating alkyl chains enhance the electron density at the nitrogen.^[^ ^23^, ^24^ ^]^ These results provide evidence supporting the presence of the 1‐*n*‐alkylimidazolium ionomers at the top layer. Using these characterization techniques, we confirm that all electrodes have similar morphologies and surface chemical states.

**Figure 3:**
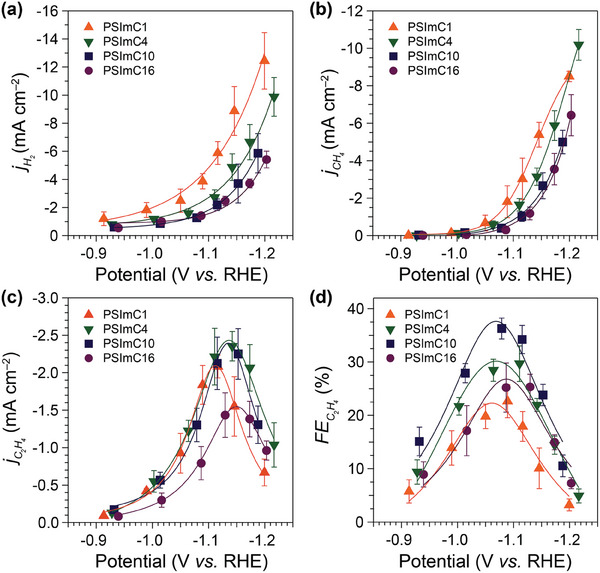
Catalytic activity and selectivity of the electrodes coated with Cu NPs and 1‐*n*‐alkylimidazolium ionomers. a–c) Partial current densities for each major product (H_2_, CH_4_, and C_2_H_4_) during electrochemical CO_2_ reduction at different applied potentials in a CO_2_‐saturated 0.1 M KHCO_3_. d) Faradaic efficiency for C_2_H_4_ measured after CO_2_ reduction for 1 h. The data reported are an average of at least four different measurements on freshly prepared samples, and the error bar represents the standard deviation.

The effect of these ionomers on the Cu‐catalyzed CO_2_RR was investigated in a two‐compartment electrochemical cell (H‐cell, Figure S12, Supporting Information) using an aqueous electrolyte of CO_2_‐saturated 0.1 M KHCO_3_. The CO_2_RR experiments were performed by chronoamperometry for 60 min at different applied potentials from –0.9 to –1.2 V versus reversible hydrogen electrode (RHE; all potentials are given with respect to RHE) and repeated at least four times using freshly prepared electrodes (Figure S13, Supporting Information). H_2_, CH_4_, and C_2_H_4_ are identified as major products, while trace amounts of CO and HCOO^−^ are detected as minor products. Detailed experimental procedures can be found in the Supporting Information. SEM was initially employed to examine potential morphology changes during CO_2_RR, and no significant changes were observed on the electrodes after an hour of electrolysis (Figure S8f–i, Supporting Information). The total current density was then plotted for each electrode as a function of applied potential (Figure S14a, Supporting Information). Interestingly, this plot shows a decrease in total current density with an increase in the side chain length of the ionomers. To better elucidate the Cu‐catalyzed CO_2_RR kinetics, the total current density was further partitioned into partial current densities for the major products (**Figure** **3**a–c) and minor products (Figure S14b,c, Supporting Information).

The electrokinetics for both HER and CH_4_ production are suppressed with the increasing side chain length of the ionomers, aligning with the trend observed in the total current densities. For example, Cu–PSImC16 shows a substantial decrease in $j_{H_{2}}$ by 56.5% at –1.20 V, from –12.4 to –5.4 mA cm^−2^, compared to Cu–PSImC1 (Figure 3a). These results are consistent with the previous report indicating that bulkier substituents on an imidazolium ionomer modulate the promotion of HER during Ag‐catalyzed CO_2_RR.^[^ ^20^ ^]^ Similarly, the $j_{{\mathrm{CH}}_{4}}$ exhibits a sharp 60.0% reduction when comparing Cu–PSImC1 to Cu–PSImC16, shifting from –3.0 to –1.2 mA cm^−2^ at –1.13 V (Figure 3b).

The kinetics of the C_2_H_4_ formation reaction show a rather distinct trend compared to those of HER and CH_4_ production. The 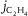 of every sample peaks at around –1.10 to –1.15 V. The peak of 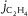 increases by 13.0% from PSImC1 to PSImC4, plateaus at PSImC4 and PSImC10, and then decreases by 36.2% from PSImC10 to PSImC16 (Figure 3c). Again, the kinetics for H_2_ and CH_4_ production decrease with longer alkyl side chains, while those for C_2_H_4_ production exhibit an optimal range across the samples. This phenomenon leads to a general trend of larger $FE_{C_{2}H_{4}}$ with PSImC10 compared to that with PSImC4, showing that the selectivity for C_2_H_4_ is optimized with PSImC10 among the series of ionomers (Figure 3d). As a result, the maximum $FE_{C_{2}H_{4}}$ with PSImC10 is determined to be 36.3% at –1.08 V, whereas that with PSImC1 is only 22.7% at –1.09 V. These data emphasize a remarkable 59.9% increase in $FE_{C_{2}H_{4}}$ solely by increasing the number of carbons in the alkyl chain from one to ten. The *FE*s for H_2_, CH_4_, CO, and HCOO^−^ over the range of applied potential are shown in Figure S15 (Supporting Information).

**Figure 4:**
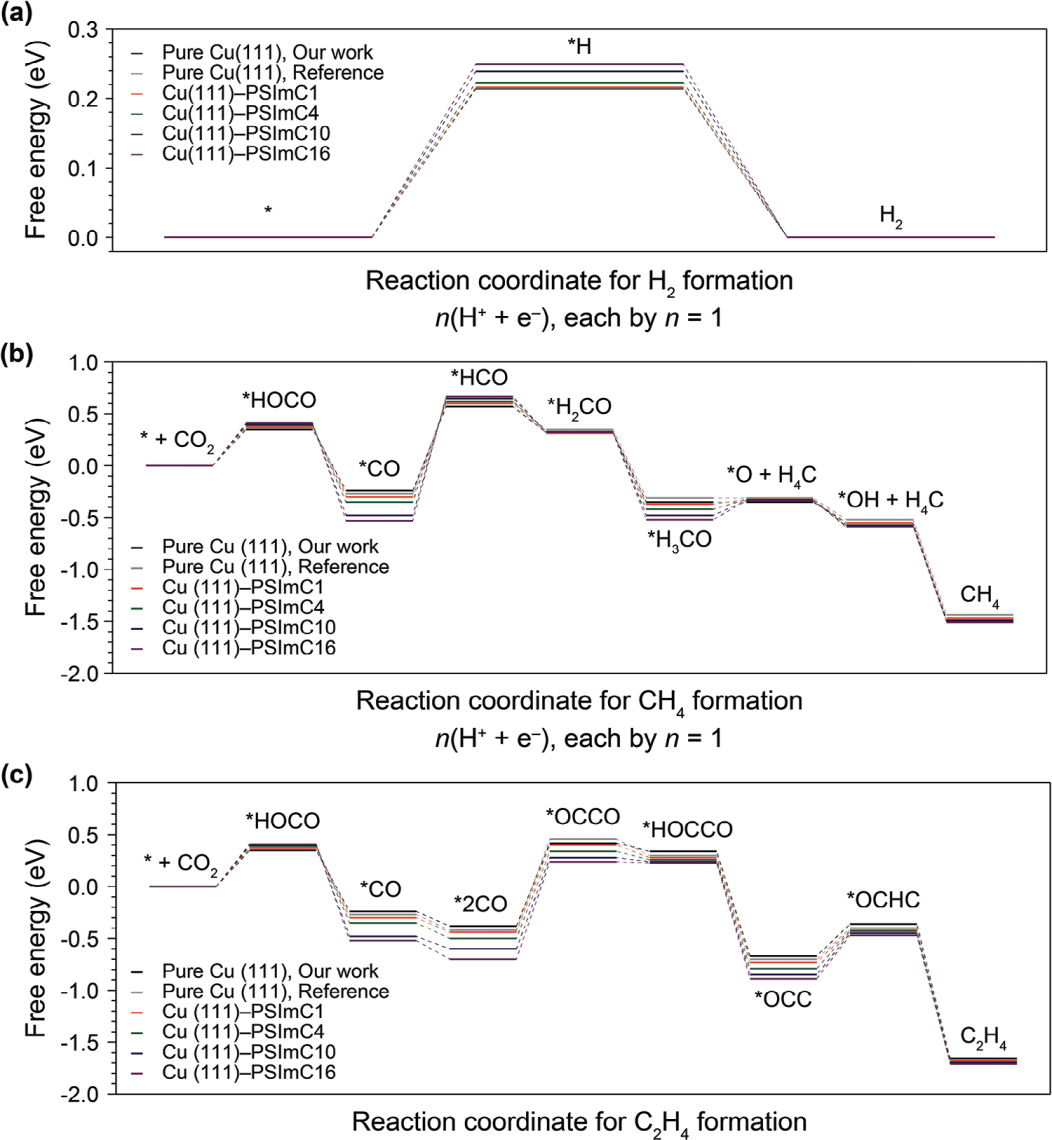
Free energy diagram (in eV) for the formation reactions of a) H_2_, b) CH_4_, and c) C_2_H_4_, with various alkyl chain lengths of ionomers (PSImC1, PSImC4, PSImC10, and PSImC16) on the Cu(111) surface, computed from DFT calculations. The same calculations without the ionomers (Pure Cu(111)) are also compared with those from the references.^[^ ^25^, ^26^ ^]^ The rate‐determining steps (RDS) and the corresponding intermediate states for each case are chosen from the references.^[^ ^25^, ^26^ ^]^ For the H_2_ and CH_4_ formations, the reaction coordinate describes a proton‐coupled electron transfer at each step (i.e., *n*(H^+^ + e^−^) with *n* = 1). For the C_2_H_4_ formation, the reaction coordinate follows the RDS identified from the reference.^[^ ^27^ ^]^

DFT calculations were conducted to develop a more thorough understanding of the thermodynamics and kinetics of CO_2_RR pathways on ionomer‐coated Cu NP electrodes (**Figure** **4**). Directly computing the entire structures of 1‐*n*‐alkyl‐3‐(4‐vinylbenzyl)imidazolium moieties with repetitive polymeric backbones using a periodic plane‐wave basis DFT is nearly infeasible due to computational constraints. We thus opted for a simplified model using the 1‐*n*‐alkyl‐3‐methylimidazolium series with varying alkyl chain lengths to approximate the complexity of the original molecules. For consistency throughout the paper and for ease of reference, the same nomenclature as the experimental imidazolium moieties (i.e., PSImC1, PSImC4, PSImC10, and PSImC16) has been adopted for these simplified series. This adjustment allows us to conduct our analysis within the computational limits while maintaining relevance to the experimental design. The more detailed chemical structures of the truncated moieties, the system setup, and the computational methods for the DFT calculations are available in the Supporting Information (Figures S16–S18). Free energy pathways for the CO_2_RR with the key intermediates for the H_2_, CH_4_, and C_2_H_4_ formation reactions on the Cu(111) slab with the series of 1‐*n*‐alkyl‐3‐methylimidazoliums are shown in Figure 4a–c, respectively. Here, only the RDS, including the key intermediates from the literature, is considered for each formation reaction.^[^ ^5^, ^25^, ^26^, ^27^, ^28^ ^]^

For the HER, the increase in alkyl side chain length leads to higher activation energy required to convert H^+^ ions into the *H adsorbate, the sole intermediate (Figure 4a). The calculation results closely align with the experimental results (Figure 3a), demonstrating that HER is suppressed with increasing side chain lengths of the ionomers. It is worth noting that the presence of imidazolium moieties suppresses the overall HER formation, compared to the bare Cu(111) surface without the moieties,^[^ ^25^ ^]^ by destabilizing the free‐energy state of the *H adsorbate. This suggests that the presence of imidazolium moieties and the larger steric bulk of the longer alkyl side chains may gradually hinder the interaction between the Cu(111) surface and H^+^ ions, which in turn could reduce the surface coverage of the *H adsorbate and slow the kinetics of the HER.

For the CH_4_ formation reaction, the DFT calculations (Figure 4b) also support the electrochemical measurements (Figure 3b). With the increasing side chain length, the key *CO intermediate of the RDS (i.e., *CO → *HCO) is significantly stabilized due to its lower free energy state, while the *HCO intermediate is slightly destabilized due to the higher free energy state. Together, this trend suggests that the increase in the side chain length leads to the higher activation energy of the proton‐coupled electron transfer step (PCET), thereby hindering the overall kinetics of the CH_4_ formation (Figure S19a, Supporting Information), as consistently observed in the experiments. It can be inferred that the PCET of the *CO intermediate to *HCO turns energetically unfavorable as the Cu(111) surface becomes less accessible due to the larger degrees of steric hindrance from the bulkier imidazolium moieties with longer side chains, thereby hindering the overall CH_4_ formation reaction (Figure 4b).

On the contrary, for the C_2_H_4_ production, the calculated activation energy for the RDS, the dimerization of *2CO to *OCCO, shows negligible differences across the samples (Figure S19b, Supporting Information). The relative kinetics are thus evaluated based on the relative free energy level of the *2CO intermediate. Figure 4c reveals that the *2CO state becomes more stabilized with increasing alkyl chain length, as evidenced by a lower free energy level, implying more facile reaction kinetics for C_2_H_4_ formation. Potential‐dependent in situ Raman spectroscopy was subsequently performed on the representative PSImC1 and PSImC10 samples to substantiate our calculations experimentally (see Supporting Information for details). Both samples exhibit a monotonic increase in the intensity of the C–O vibration peak in the 1900–2100 cm^−1^ region as the potential is increased from the open circuit potential (OCP) to –0.9 V. From –1.0 to –1.2 V, however, the intensity begins to decrease, likely due to the emergence of larger gas bubbles at the electrode‐electrolyte interface (Figure S21a, Supporting Information). This CO vibration peak at –0.9 V was thus deconvoluted into three distinct components: bridged CO, low‐frequency band (LFB) CO, and high‐frequency band (HFB) CO (Figure S21b, Supporting Information). Recent work by An et al. reported that the dynamic CO intermediate in the LFB region (below 2060 cm^−1^) is associated with C–C coupling and C_2_H_4_ formation, while the static HFB CO intermediate is correlated with CO production.^[^ ^29^ ^]^ Our in situ Raman measurements corroborate the significant role of the LFB CO intermediate in C_2_H_4_ formation. Specifically, the high ratio of the integrated area between the LFB and HFB peaks for PSImC10 suggests that C–C coupling and C_2_H_4_ production are more favorable on PSImC10 compared to PSImC1 (Figure S21c, Supporting Information). Both the computational and experimental findings elucidate important details on the preferred formation of C_2_H_4_ with longer alkyl side chains of the imidazolium. These results align with the electrokinetic trend in the range of PSImC1 to PSImC4, where 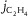 increases; however, the same trend is not observed in the range of PSImC4 to PSImC16.

**Figure 5:**
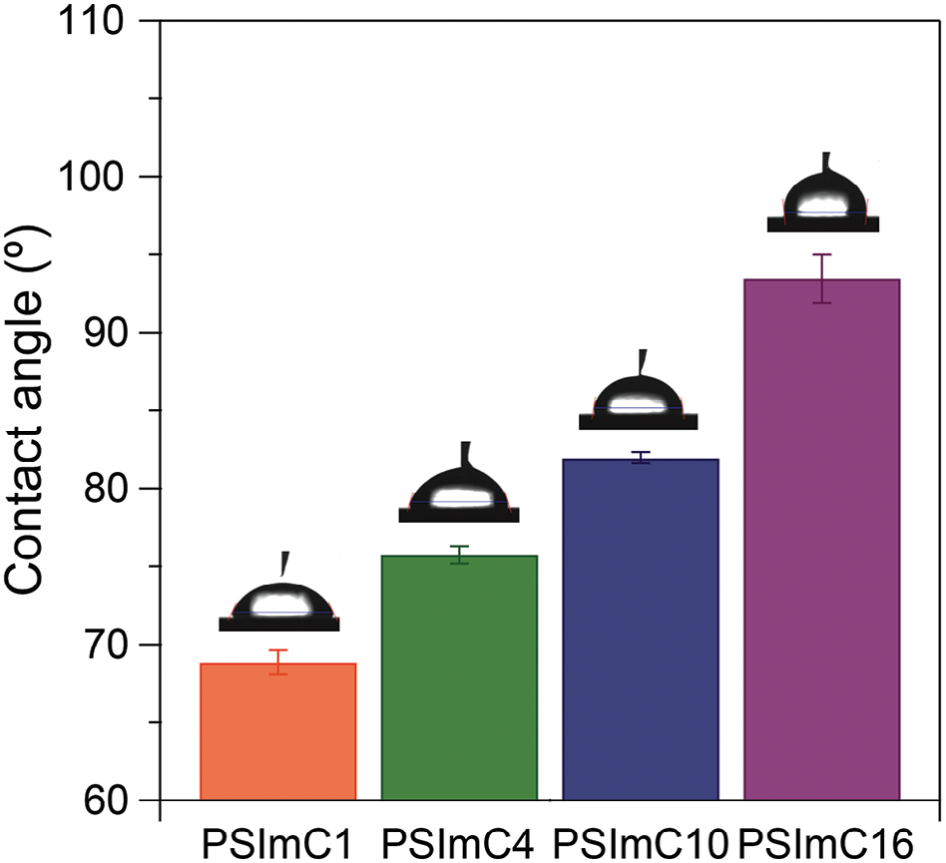
Water contact angle measurements on the as‐prepared electrodes coated with the Cu NPs and 1‐*n*‐alkylimidazolium ionomers. The contact angle increases with the alkyl side chain length.

Both the hydrophobicity of electrodes and the ionic conductivity of ionomers were subsequently assessed to complement the insights from the DFT calculations across the entire range of alkyl side chain lengths. The hydrophobicity of the as‐prepared electrodes, which were coated with Cu NPs and ionomers, was characterized by measuring the contact angle of water droplets on them (**Figure** **5**). Notably, the water contact angle gradually increases from 69° to 93° with the increasing length of the alkyl side chains. The gradual increase in contact angle is attributed to the incorporation of longer and more hydrophobic alkyl segments into the ionomers. These contact angle measurements are in line with the electrochemical CO_2_RR results (Figure 3a,b) and DFT calculations (Figure 4a,b) that demonstrate the suppression of both H_2_ and CH_4_ formation reactions. A more hydrophobic environment near the catalyst surface, created by an ionomer with longer side chains, leads to a lower local concentration of H_2_O while simultaneously increasing those of CO_2_ and the *CO intermediate.^[^ ^10^ ^]^ The lower local concentration of H_2_O decreases the availability of the *H adsorbate, the sole intermediate in the HER, which in turn suppresses the reaction.^[^ ^30^ ^]^ The CH_4_ formation reaction is similarly suppressed in a hydrophobic environment since the hydrogenation of *CO to *HCO, which is considered to be the RDS for the reaction,^[^ ^31^ ^]^ is hindered due to the limited availability of the *H adsorbate. On the other hand, the hydrophobic catalyst surface is reported to enhance C_2_H_4_ production kinetics by limiting the water diffusion and providing a higher local pH, which subsequently promotes the C–C coupling.^[^ ^32^ ^]^

The ion‐exchange capacity (IEC) and ionic conductivity (σ) were then determined for each ionomer to expand our understanding of the 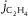 profile across the entire range of alkyl side chain lengths (see Supporting Information for details). The IEC was designed to decrease in this ionomer series with increasing alkyl side chain length (**Table** **1**). This reduction in IEC is attributed to the ionomers sharing the same parent copolymer, P(S‐*co*‐VBC), which provides a consistent molar ratio between styrene and VBC in all samples. The functionalization of the P(S‐*co*‐VBC) with 1‐*n*‐alkylimidazoles having longer side chains reduces the number of imidazolium groups per gram of ionomer and thus decreases the IEC. The reduction in IEC directly leads to lower ionic conductivity. Table 1 shows a linear relationship between these metrics, with *σ*/IEC values demonstrating remarkable consistency. This linear relationship indicates that variations in side chain length do not substantially influence ionic conductivity; instead, the decrease in ionic conductivity is primarily due to the reduction in IEC. The reduced conductivity reflects less efficient transport of OH^−^ anions from the cathode to the electrolyte.^[^ ^33^ ^]^ This inefficiency creates a microenvironment with higher interfacial resistance at the electrode‐electrolyte interface, which can adversely affect the electrokinetics of both HER and CO_2_RR. However, the complexity of C_2_H_4_ formation, which involves multi‐electron transfer processes via different active sites (e.g., C–C coupling), may make it particularly sensitive to ionic conductivity. In contrast, HER and CH_4_ formation undergo fewer elementary steps, making them likely less affected. This effect not only explains the suppression of H_2_ and CH_4_ formation kinetics with longer side chains but also elucidates rather distinct kinetics of C_2_H_4_ formation.

**Table 1:** Properties of the 1‐*n*‐alkylimidazolium ionomers synthesized for this study.

Ionomers	*M*_*n*_ [kg mol^−1^]	IEC [meq g^−1^]^a)^	Ionic conductivity, σ [mS cm^−1^]^b)^	σ/IEC [g S cm^−1^ eq^−1^]
PSImC1	22.6	2.34	0.72	0.32
PSImC4	25.0	2.13	0.66	0.31
PSImC10	29.8	1.80	0.53	0.29
PSImC16	34.6	1.57	0.46	0.30

a) IEC was determined by ^1^H NMR in chloride form;

b) Ionic conductivity was measured at 5 wt% in anhydrous DMSO.

The increase in ionomer side chain lengths divides the C_2_H_4_ production kinetics into two distinct regimes. For shorter chains (PSImC1 to PSImC4), an enhancement in the kinetics is observed as the side chain length increases. This observation is attributed to the more stable *2CO intermediate and the more hydrophobic microenvironment near the electrode that could promote the C–C coupling.^[^ ^32^ ^]^ This enhancement suggests that the moderate increase in side chain length can favorably influence the C_2_H_4_ production kinetics. Conversely, within the longer side chain region (PSImC10 to PSImC16), the kinetics begin to decelerate with further increases in side chain length, primarily ascribed to the corresponding decrease in ionic conductivity. This dual‐phase kinetic behavior underscores a complex interplay among the thermodynamic stability of the key intermediate, the tuned microenvironment near the electrode, and the ionic conductivity in determining overall kinetics. It is important to note that the kinetics improve with the side chain length up to an optimal point (PSImC10), beyond which the thermodynamic advantages are offset by the drawbacks of reduced conductivity. Our findings provide an in‐depth understanding of how variations in side chain length influence the kinetics of both CO_2_RR and HER.

**Figure 6:**
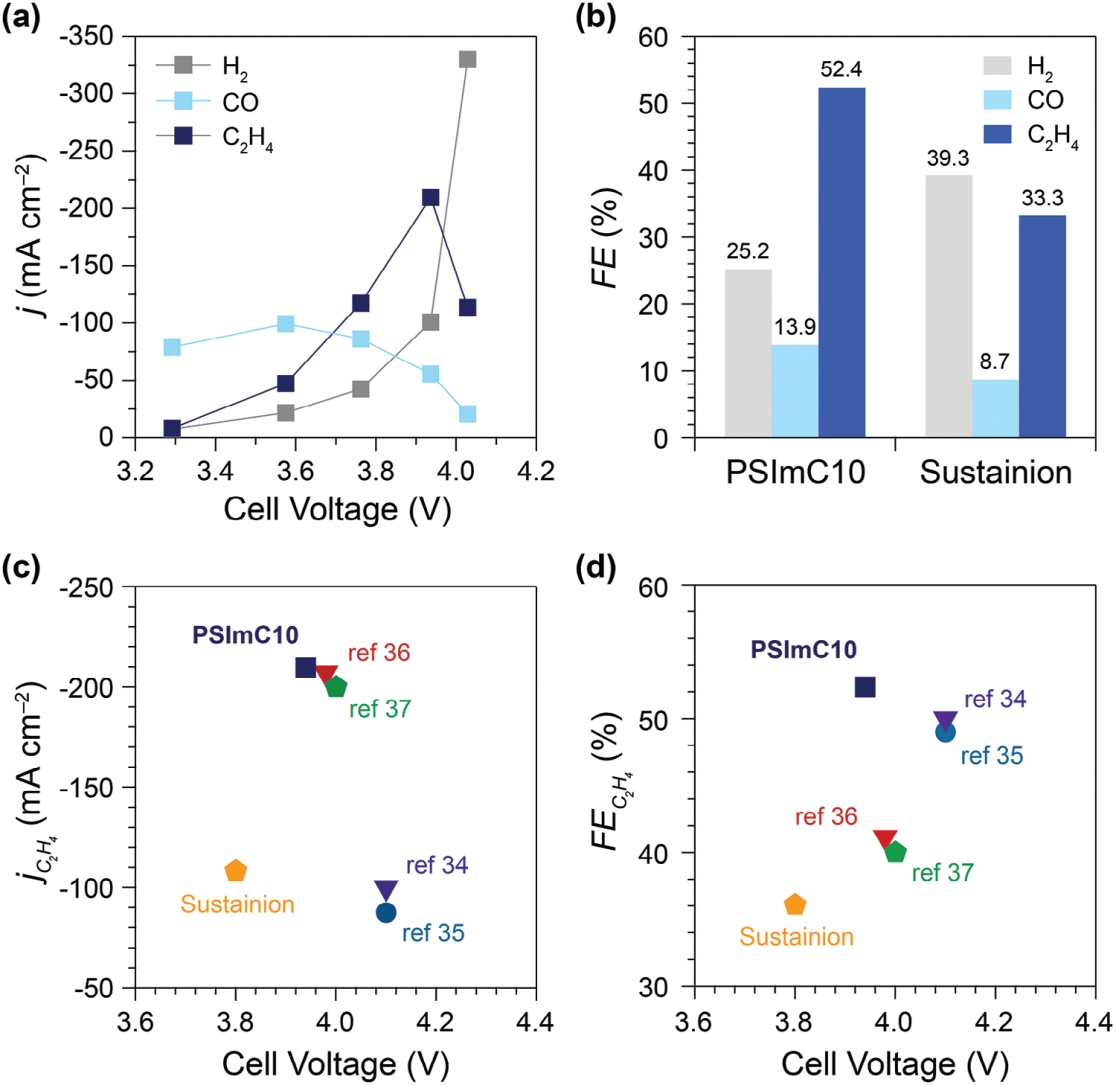
a) Partial current densities (*j*) for major products (H_2_, CO, and C_2_H_4_) with the PSImC10 ionomer in a membrane electrode assembly at various cell voltages. b) Faradaic efficiencies (*FE*) for the major products with the PSImC10 at 3.95 V compared to those with the commercial Sustainion ionomer. c,d) Comparison of PSImC10, Sustainion, and literature data on maximum (c) 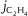 and (d) $FE_{C_{2}H_{4}}$ versus cell voltage. The results from this study are indicated as PSImC10 and Sustainion. For comparative analysis, literature data were extracted from studies using Cu catalysts of moderate catalytic activity (i.e., commercial Cu nanoparticles or sputtered Cu). Symbols: square (PSImC10), pentagon (Sustainion), inverted triangle (Nafion), and circle (PTFE).

Finally, the best‐performing PSImC10 ionomer was utilized as a binder for a commercial Cu nanopowder in a membrane electrode assembly (MEA) to demonstrate its potential for industrial applications (**Figure** **6**a; Figure S23, Supporting Information). The partial current densities for major products (H_2_, CO, and C_2_H_4_) display similar profiles as a function of cell voltage to those observed in an H‐cell (Figure 3a–c). A notable difference is the trace amount of CH_4_ produced in the MEA, in contrast to the H‐cell results. This discrepancy may be attributed to the higher local concentration of CO_2_ near the catalyst surface in the MEA, resulting in increased surface coverage of CO_2_ and decreased selectivity to CH_4_ in CO_2_RR.^[^ ^34^ ^]^ The selectivity using PSImC10 was then compared with that using the commercial Sustainion ionomer across various cell voltages (Figure 6b; Figure S23, Supporting Information). PSImC10 exhibits greater suppression of the HER and enhanced promotion of C_2_H_4_ production, leading to higher $FE_{C_{2}H_{4}}$ across the voltage range. The 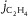 reaches –209.5 mA cm^−2^ at a cell voltage of 3.95 V (Figure 6a). Furthermore, the $FE_{C_{2}H_{4}}$ with PSImC10 achieves a maximum of 52.4% at 3.95 V, compared to 33.3% with Sustainion, marking a significant 57.4% increase in $FE_{C_{2}H_{4}}$ (Figure 6b). Comparative analysis was conducted using data from the literature focusing on studies with Cu catalysts of moderate catalytic activity (i.e., commercial Cu nanoparticles or sputtered Cu).^[^ ^35^, ^36^, ^37^, ^38^ ^]^ While engineering MEA electrolyzers to enhance cell performance is beyond the scope of this study, it is important to note the potential for further improvements by exploring ionomer moieties, employing more effective catalysts, and optimizing reactor designs. Nevertheless, by solely adjusting the alkyl side chain length in the ionomers, this operation with the PSImC10 achieved even higher $FE_{C_{2}H_{4}}$ of 52.4% and 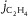 of –209.5 mA cm^−2^ at a low cell voltage of 3.95 V, surpassing the previous benchmarks (Figure 6c–d).

## 3 Conclusion

In summary, we investigated the impact of the side chains in 1‐*n*‐alkylimidazolium ionomers on Cu‐catalyzed CO_2_RR. The production rates of both H_2_ and CH_4_ decline as the side chain length increases, with the PSImC16 showing the most suppressed reaction rates by up to 56.5% and 60.0%, respectively. On the other hand, the C_2_H_4_ production kinetics increase, plateau, and then decrease with increasing side chain lengths. Due to these different kinetic behaviors of the major products, the C_2_H_4_ production demonstrates an optimal Faradaic efficiency of 36.3% with the PSImC10, a substantial increase of 59.9% compared to that with the PSImC1. The integrative approach of combining DFT calculations, in situ Raman spectroscopy, hydrophobicity, and ionic conductivity assessments elucidates how side chain optimization can precisely modulate the energetic stability of the key intermediates (i.e., *H, *CO, *HCO, and *2CO), thereby influencing the selectivity of both CO_2_RR and HER. This work provides a proof‐of‐principle demonstration that ionomer engineering can achieve an industrially relevant 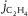 of –209.5 mA cm^−2^ with a remarkably high $FE_{C_{2}H_{4}}$ of 52.4% at a relatively low voltage of 3.95 V, outperforming previous literature data. Our study also underscores the importance of systematic studies on the structural moieties of ionomers for CO_2_RR targeting specific products. Further investigations are now expanding to comprehensively examine how variations in ionomer structure beyond side chain length can enhance our understanding and control over CO_2_RR processes.

## Conflict of Interest

The authors declare no conflict of interest.

## Supporting information

Supporting Information

## Data Availability

The data that support the findings of this study are available from the corresponding author upon reasonable request.
