# Supplementary material for: Impact of Side Chains in 1‐n‐Alkylimidazolium Ionomers on Cu‐Catalyzed Electrochemical CO2 Reduction
Source: Adv Sci (Weinh). 2024 Oct 31;11(47):2406281. doi: 10.1002/advs.202406281 (PMC11653625; doi:10.1002/advs.202406281)
Supplement: Supplementary file 1 — Supporting Information [file ADVS-11-2406281-s001.pdf]

ADVANCED SCIENCE
Open Access

Supporting Information

for *Adv. Sci.*, DOI 10.1002/advs.202406281

Impact of Side Chains in 1-*n*-Alkylimidazolium Ionomers on Cu-Catalyzed Electrochemical CO_2 Reduction

Young In Song, Bohak Yoon, Chanwoo Lee, Dogyeong Kim, Man Ho Han, Hyungu Han, Woong Hee Lee, Da Hye Won, Jung Kyu Kim, Hyo Sang Jeon* and Jai Hyun Koh**

Supporting Information for

Impact of Side Chains in 1-*n*-Alkylimidazolium Ionomers on Cu-Catalyzed Electrochemical CO_2 Reduction

Young In Song,[1,2,†] Bohak Yoon,[3,†] Chanwoo Lee,[1,4] Dogyeong Kim,[1,5] Man Ho Han,[1] Hyungu Han,[2] Woong Hee Lee,[1,4] Da Hye Won,[1,4] Jung Kyu Kim,[2,*] Hyo Sang Jeon,[6,*] and Jai Hyun Koh[1,4,*]

[†]These authors contributed equally to this work.

[1]Clean Energy Research Center, Korea Institute of Science and Technology (KIST), Seoul 02792, Republic of Korea

[2]School of Chemical Engineering, Sungkyunkwan University (SKKU), Suwon 16419, Republic of Korea

[3]Department of Chemistry, Chicago Center for Theoretical Chemistry, Institute for Biophysical Dynamics, and James Franck Institute, The University of Chicago, Chicago, IL 60637, USA

[4]Division of Energy & Environment Technology, KIST School, University of Science and Technology, Seoul 02792, Republic of Korea

[5]Department of Chemical and Biological Engineering, Korea University, Seoul 02841, Republic of Korea

[6]Technological Convergence Center, Korea Institute of Science and Technology (KIST), Seoul 02792, Republic of Korea

Corresponding Authors

*Email: legkim@skku.edu (J.K.K.); hsjeon@kist.re.kr (H.S.J.); jhkoh@kist.re.kr (J.H.K.)

Table of Contents

1. Experimental **3**

1.1. Materials 3

1.2. RAFT Copolymerization of Styrene and 4-Vinylbenzyl Chloride 3

1.3. Removal of Trithiocarbonyl End-group of RAFT-polymerized P(S-*co*-VBC) 3

1.4. Synthesis of 1-*n*-Alkylimidazoles 3

1.4.1 1-*n*-Butylimidazole 3

1.4.2 1-*n*-Decylimidazole 4

1.4.3 1-*n*-Hexadecylimidazole 4

1.5. Functionalization of Styrene-based Copolymers with 1-*n*-Alkylimidazoles 4

1.6. Preparation of Cu Nanoparticle (NP) Electrodes 4

1.7. Electrochemical CO_2RR 4

1.7.1 Two-compartment Electrochemical Cell 4

1.7.2 Membrane Electrode Assembly 5

1.8. Ionic Conductivity Measurements 5

1.9. Ion-exchange Capacity (IEC) 6

1.10. ^{1}H NMR Spectroscopy 6

1.11. FT-IR Spectroscopy 6

1.12. Gel Permeation Chromatography (GPC) 6

1.13. Differential Scanning Calorimetry (DSC) 6

1.14. Thermogravimetric Analysis (TGA) 6

1.15. X-ray Diffraction (XRD) 6

1.16. X-ray Photoelectron Spectroscopy (XPS) 7

1.17. Scanning Electron Microscopy (SEM) 7

1.18. Water Contact Angle (CA) Analysis 7

1.19. In-situ Surface-enhanced Raman Spectroscopy (In-situ SERS) 7

2. Density Functional Theory (DFT) Calculations **7**

3. Supporting Figures and Tables **9**

4. References **27**

1. Experimental

Materials. Styrene (≥99%), 4-vinylbenzyl chloride (VBC, 90%), imidazole (≥99%), 2-cyano-2-propyl dodecyl trithiocarbonate (CPDT), lauroyl peroxide (LPO), toluene (anhydrous, 99.8%), 1-methylimidazole (99.8%), 1-bromobutane (99%), 1-bromodecane (99%), 1-bromohexadecane (97%), potassium hydroxide (90%, flakes), diethyl ether (≥99%), copper (nanopowder, 25 nm particle size) and potassium bicarbonate (99.7%) were purchased from Sigma-Aldrich. Aluminum oxide (5016-A, Basic, Brockmann Grade I), magnesium sulfate (anhydrous, 99.5% min), and glassy carbon substrate were purchased from Alfa Aesar. Tetrahydrofuran (THF, 99.5%), *N,N*-dimethylformamide (DMF, HPLC, 99.8%), methanol (99.5%), ethyl acetate (EtOAc, 99%), 2,2–azobisisobutyronitrile (AIBN, 99%) were purchased from Daejung Chemicals. AIBN and LPO were recrystallized from methanol and diethyl ether, respectively, prior to use. All chemicals and solvents were used without further purification unless stated otherwise.

RAFT Copolymerization of Styrene and 4-Vinylbenzyl Chloride. The stabilizers in styrene and VBC were removed by passing through a basic alumina column prior to polymerization. The copolymer of styrene and VBC was synthesized by RAFT polymerization using AIBN and CPDT as the initiator and chain transfer agent, respectively. Inhibitor-free styrene (15.80 g, 151.70 mmol, 0.70 eq.), VBC (9.92 g, 65.00 mmol, 0.30 eq.), AIBN (4.7 mg, 0.03 mmol, 0.13 meq.), and CPDT (98.8 mg, 0.29 mmol, 1.32 meq.) were added to a 100 mL two-neck round-bottom flask charged with a magnetic stir bar and a rubber septum. The reaction mixture was vigorously purged with argon for 30 min and then heated at 70 ℃ for 24 h. The reaction mixture was then quenched at 0 ℃ and precipitated into methanol. The crude polymer was isolated by filtration, dissolved in THF, and re-precipitated in methanol two more times to remove any remaining monomers and impurities. The purified polymer was dried *in vacuo* at 60 ℃ to yield 6.51 g (25.2%) of poly(styrene-*co*-4-vinylbenzyl chloride), P(S-*co*-VBC), as a yellow powder.

Removal of Trithiocarbonyl End-group of RAFT-polymerized P(S-*co*-VBC). The CPDT end-group ($C_{12}H_{25}S_2CS$–) of the copolymer was removed by a procedure from the literature using a mixture of AIBN and LPO.[1] The purpose of this procedure was to prevent unwanted chain transfer reactions that can be caused by radicals generated from the CPDT end-groups during the subsequent functionalization reactions with 1-*n*-alkylimidazoles. The end-group of P(S-*co*-VBC) was removed by heating a mixture of the copolymer (6.51 g, 0.31 mmol, 1.0 eq.), AIBN (1.00 g, 6.11 mmol, 20.0 eq.), and LPO (0.24 g, 0.61 mmol, 2.0 eq.) in 10 mL of toluene at 80 ℃ for 4 h. The resulting product was obtained as a white powder using the same precipitation procedure described above.

Synthesis of 1-*n*-Alkylimidazoles. A series of 1-*n*-alkylimidazoles, except for the purchased 1-methylimidazole, was synthesized using a slightly modified procedure from the literature.[2] The synthetic procedure for 1-*n*-butylimidazole is outlined as a representative example of the series.

1-n-Butylimidazole. A three-neck round-bottom flask equipped with a condenser, a rubber septum, and a stir bar was flame-dried under vacuum. Imidazole (3.00 g, 44.07 mmol, 1.00 eq.), potassium hydroxide (2.97 g, 52.88 mmol, 1.20 eq.), and dry DMF (30 mL) were added to the flask under argon with vigorous stirring. 1-Bromobutane (6.40 g, 46.71 mmol, 1.06 eq.) was then added dropwise to the flask and heated at 120 ℃ for 16 h. The reaction mixture was then quenched with water (200 mL) at 0 ℃. The solution was extracted with DCM (3 × 100 mL). The combined organic layers

were washed with water (2 × 200 mL) and brine (1 × 200 mL). The organic layer was dried over magnesium sulfate. The remaining solvent was removed by rotary evaporation and the product was further dried *in vacuo*. Silica gel chromatography (EtOAc:MeOH = 9:1) was used to yield 1.40 g (25.5%) of the product as a pale yellow oil.

1-n-Decylimidazole. 1-Bromodecane was used instead of 1-bromobutane. The rest of the procedure was the same as described above. 1-*n*-Decylimidazole was obtained as a very pale yellow oil.

1-n-Hexadecylimidazole. 1-Bromohexadecane was used instead of 1-bromobutane. The rest of the procedure was the same as described above. 1-*n*-Hexadecylimidazole was obtained as a white powder.

Functionalization of Styrene-based Copolymers with 1-*n*-Alkylimidazoles. A 20-mL scintillation vial equipped with a magnetic stir bar and a rubber septum was flame-dried and charged with P(S-*co*-VBC) (1.3 g, 0.06 mmol, 1.0 eq.), 1-*n*-alkylimidazole (5.7 mmol, 100.0 eq.), and dry DMF (15 mL). The solution was stirred at room temperature for 30 min and then heated at 110 ºC for 48 h. The solution was precipitated into diethyl ether, and the resulting brown powder was dried in vacuo at 60 ºC. The total recovered mass was 1.63 g (82%).

Preparation of Cu Nanoparticle (NP) Electrodes. For the cathode, the 1:1 ratio of Cu NPs and as-synthesized ionomer mixture was dispersed in methanol by sonication for 30 min to form the catalyst ink. The ink was then cast onto a glassy carbon substrate (0.5 cm^2) with a respective loading of 0.1 $mg\,cm^{-2}$ on a hot plate at 60 ºC. These Cu NP electrodes were used as working electrodes for electrochemical CO_2 reduction reaction (CO_2RR) in a two-compartment electrochemical cell.

Electrochemical CO_2RR. Electrochemical CO_2RR experiments were conducted either in a two-compartment electrochemical cell or a membrane electrode assembly (MEA), depending on the purposes. The two-compartment electrochemical cell (Figure S12) was used to examine the impact of the side chains in the ionomers on the catalytic activity and selectivity. The membrane electrode assembly was employed to demonstrate a proof-of-concept that the ionomers used in this study can achieve industrially relevant current densities with reasonably high $FE_{C_2H_4}$.

Two-compartment Electrochemical Cell. Two electrolyte chambers (38 mL each, 0.1 M $KHCO_3$) were separated by an anion-exchange membrane (Selemion AMV; AGC). The cell employed three electrodes: an ionomer-coated Cu/glassy carbon working electrode, a leak–free Ag/AgCl reference electrode (3.4 M KCl, Innovative Instrument), and a Pt mesh counter electrode (Qrins). The electrolyte was 0.1 M $KHCO_3$ solution being purged with CO_2 at a flow rate of 20 sccm, controlled by a mass flow controller (Bronkhorst). Electrochemistry was performed using a potentiostat (IviumStat). All potentials were converted to the RHE scale as follows:

$$E_{RHE} = E_{Ag/AgCl} + 0.242\text{ V} + 0.059\text{ V} \times \text{pH}.$$

A typical constant-potential electrolysis of CO_2 was conducted for 60 min. The steady-state current density was plotted against the applied potential. Solution resistance (R_s) was measured by electrochemical impedance spectroscopy prior to each CO_2RR experiment. The potential converted to the RHE scale was then corrected for ohmic losses (iR_s). The liquid products were quantitatively analyzed by ^{1}H NMR as described in the literature.[3] The gas products in the headspace

of the electrochemical cell were quantitatively analyzed by a gas chromatography (7890B, Agilent Technologies) equipped with a flame ionization detector (FID) and a thermal conductivity detector (TCD). The faradaic efficiency (*FE*) of a gas product, *i*, was calculated by integrating the areas of the corresponding peaks in a GC chromatogram as shown below:

$$FE_i = V_i \times \text{flow rate} \times \frac{nFp_0}{RT_0 i_{total}}.$$

where V_i is the volume concentration of the product, *i*, based on the calibration of the GC, the flow rate is measured by a universal flow meter (Agilent) at the exit of the electrochemical cell, n is the number of transferred electrons for a specific product, i_{total} is a total steady–state current, F = 96485 C mol^{-1}, p_0 = 1.013 bar, T_0 = 298.15 K, and R = 8.314 J mol^{-1} K^{-1}.

Membrane Electrode Assembly. A Cu catalyst ink was formulated by dispersing 30 mg of copper nanopowder (Chempur) with a 5 wt% ionomer solution in 1 ml of IPA via sonication. The cathode was then prepared by spray-coating the Cu catalyst ink onto a gas-diffusion electrode (GDE) at 70°C, with a catalyst loading of 1.0 mg cm^{-2}. Electrochemical experiments were carried out using a 10 cm^2 membrane electrode assembly (MEA). A Sustanion® membrane (X37-50 grade 60, Dioxide Materials) was activated in a 1 M KOH solution at 55 ºC for 3 h, followed by gentle rinsing with DI water prior to use. The membrane was then placed between the anode and cathode plates for the MEA. Humidified gaseous CO_2 was fed to the cathode plate at 200 sccm at 55 ºC, while a 0.1 M $KHCO_3$ solution was supplied to the anode plate at various flow rates using a pump. Each electrochemical experiment was conducted by employing chronopotentiometry for 24 min with a VSP potentiostat (Biologic). Electrochemical impedance spectroscopy (EIS) was performed at a current density of 300 mA cm^{-2} over the frequency range of 0.1 Hz to 100 kHz. The outlet gas mixture through a water trap was quantitatively analyzed using gas chromatography (7890B, Agilent Technologies) equipped with an FID and a TCD.

It is important to note that the MEA experiments were conducted with a Cu catalyst loading of 1.0 mg cm^{-2}, which is ten times higher than the 0.1 mg cm^{-2} used for the initial H-cell experiments. This higher catalyst loading for the MEA was intended to achieve industrially relevant performance by leveraging the benefits of the MEA setup, in which the mass transport of gaseous CO_2 is significantly improved compared to the H-cell. In the H-cell, the partial current densities for both hydrogen evolution reaction (HER) and C_2H_4 production increase when the higher Cu loading of 1.0 mg cm^{-2} was employed (Figure S24). However, HER exhibited a more pronounced increase compared to C_2H_4 formation. This difference is likely due to the mass transport limitation of aqueous CO_2 in the H-cell, where the solubility of CO_2 is limited to 34 mM at room temperature. We thus conclude that the Cu loading of 0.1 mg cm^{-2} is more appropriate under the specific conditions in the H-cell, whereas the higher loading of 1.0 mg cm^{-2} is beneficial for achieving higher current densities with improved faradaic efficiencies in the MEA setup.

Ionic Conductivity Measurements. The ionic conductivity (σ) of an ionomer in chloride form was measured by electrochemical impedance spectroscopy (EIS, Biologic VMP3B-10) using a Swagelok-type cell with two stainless steel electrodes. The loading weight of the ionomers was consistently maintained across all samples to mirror the electrode conditions in CO_2RR. Typically, an ionomer (58 mg) was dissolved in anhydrous DMSO (1.1 g) at 5 wt% concentration. The resulting ionomer solution was then loaded in the Swagelok-type cell to measure the solution resistance (R_s) by EIS. EIS was performed in a frequency range from 1 MHz to 5 Hz at a voltage amplitude of

10 mV. The solution resistances were obtained from the x-intercept from the Nyquist plots (Figure S22). The ionic conductivity in chloride form was calculated using the following equation:

$$\sigma = \frac{L}{R_s \times A}$$

where L (cm) is the distance between the two electrodes of the Swagelok-type cell, and A (1.99 cm^2) is the cross-sectional area of each electrode.

Ion-exchange Capacity (IEC). The IEC of an ionomer was calculated using the following equation:

$$IEC = \frac{1-x}{xFW_x + (1-x)FW_y} \times 1000\ (\mathrm{meq\,g^{-1}})$$

where x is a mole fraction of the styrene monomer in an ionomer, and FW_x and FW_y are the formula weight of styrene and 1-*n*-alkyl-3-(4-vinylbenzyl)imidazolium chloride, respectively. For example, the IEC of the PSImC10 was calculated as 1.80 meq g^{-1} using x of 0.65, FW_x of 104.15 g mol^{-1}, and FW_y of 360.97 g mol^{-1}.

^{1}H NMR Spectroscopy. All synthesized polymers and 1-*n*-alkylimidazoles were characterized by ^{1}H NMR spectroscopy at 400 MHz using a Bruker Ascend or Avance III HD spectrometer. The polymers were dissolved in deuterated chloroform ($CDCl_3$) or dimethyl sulfoxide (DMSO$-d_6$).

FT-IR Spectroscopy. Ionomers were ground into a fine powder, mixed with potassium bromide, and pressed into thin pellets. Transmittance spectra were then collected using a Vertex 80V spectrometer, with 100 scans performed at a resolution of 4 cm^{-1} over the range of 400–4000 cm^{-1}. The background signal was recorded under the same conditions but without a pellet in place.

Gel Permeation Chromatography (GPC). The molecular weight and dispersity of the P(S-*co*-VBC) were determined by GPC. The GPC data were collected using a JASCO LC-4000 Series HPLC system equipped with a JASCO RI-4030 refractive index (RI) detector and three Shodex columns (KF-802.5, KF-803, KF-804). THF was used as the eluent at 40 °C with a flow rate of 1.0 mL min^{-1}. The column set was calibrated with polystyrene standards.

Differential Scanning Calorimetry (DSC). DSC was performed using a TA Instruments DSC Q200 equipped with an SP Industries FC100 cooling system. The measurements were performed in a temperature range from 40 °C to 200 °C at a heating rate of 10 °C min^{-1} under N_2. The T_gs were determined at the midpoints of the heat flow changes during the third cycle.

Thermogravimetric Analysis (TGA). TGA was performed using a TA Instruments SDT-Q600 under N_2. The samples were heated from room temperature to 800 °C at a ramp rate of 10 °C min^{-1}.

X-ray Diffraction (XRD). The crystal structure of the commercial Cu NPs was investigated by an XRD (D8 Advance/Lynxeye) using radiation from a Cu Kα source at 40 kV and 40 mA.

X-ray Photoelectron Spectroscopy (XPS). X-ray photoelectron spectroscopy (XPS) was performed on a PHI 5000 VersaProbe (ULVAC PHI, Japan) with Al Kα radiation. C 1s photoelectron peak (284.03 eV) was used for the calibration of the binding energy scale. All of the spectra were normalized to the maximum. Deconvolution of the XPS spectra was performed using the CasaXPS software with a Shirley baseline subtraction.

Scanning Electron Microscopy (SEM). The surface morphology and elemental composition of ionomer-coated Cu NP electrodes were investigated by an SEM (Inspect F or Inspect F50) equipped with an EDS at an acceleration voltage of 15.0 kV and a typical working distance of 10.0 mm.

Water Contact Angle (CA) Analysis. Contact angles of water droplets were measured on ionomer-coated Cu/glassy carbon electrodes using a microscope-coupled goniometer (Phoenix 150, SEO).

In-situ Surface-enhanced Raman Spectroscopy (In-situ SERS). In-situ SERS was conducted with a Raman spectrometer (Renishaw, InVia Reflex) coupled with an optical microscope. A near-infrared laser (λ = 785 nm, grating 1200 and 1800 lines mm^{-1}) were used as excitation sources. Raman scattering signals were collected using a water immersion objective (Leica Microsystems, 63$\times$, numerical aperture of 0.9). The objective was protected from the electrolyte by a Teflon (FEP) film (Goodfellow, film thickness of 0.0125 mm), wrapped around the objective. Electrochemical measurements were performed in a homemade spectro-electrochemical Raman cell made of PEEK, controlled by a potentiostat (CompactStat, Ivium Technologies). The cell featured a leak-free Ag/AgCl reference electrode positioned close to the sample and a Pt wire counter electrode.

In-situ SERS measurement was initially performed with a representative PSImC10 sample using commercial metallic Cu NPs as a catalyst. Spectra were collected from the open circuit potential (OCP) to –1.0 V vs. RHE in the cathodic potential steps, as shown in Figure S20. However, the spectra from the metallic Cu NPs showed no prominent features, except for a very small peak around 2060 cm^{-1} at –0.8 V. These Cu NPs were found unsuitable for analyzing CO_2RR intermediates due to the lack of a significant surface-enhanced Raman scattering (SERS) effect.[4]

Oxide-derived Cu NPs (OD–Cu) were thus employed as catalysts to enhance the Raman signal at the surface. These catalysts were prepared from the metallic Cu NPs following the synthetic procedure described in the literature.[5] PSImC1 and PSImC10 samples were chosen for in-situ analysis of the *CO intermediate due to their notable differences in C_2H_4 production. Spectra in the CO region (Raman shift between 1800–2400 cm^{-1}) were collected from the OCP to –1.2 V vs. RHE in the cathodic potential steps (Figure S21a). It is important to note that the CO peaks for both samples increase from the OCP to –0.9 V, and then fade from –1.0 V to –1.2 V. This behavior is likely due to the emergence of gas bubbles at the electrode surface across the higher overpotential region. The spectrum at –0.9 V for each sample was thus deconvoluted into three peaks: bridged CO, low-frequency band (LFB) CO, and high-frequency band (HFB) CO.[6] This deconvolution allowed for the comparison of the integrated area ratio between the LFB and HFB peaks (Figure S21b).

2. Density Functional Theory (DFT) Calculations

Density functional theory (DFT)[7,8] calculations are performed with the Vienna *Ab initio* Simulation Package (VASP) version 5.4.4.[9,10] The exchange-correlation functional at the level of theory of the generalized gradient approximation (GGA) with Perdew, Berke, Erzenhof (PBE) is employed.[11]

The long-range dispersion interactions are corrected by the Grimme's (D3) method[12] with Becke-Johnson (BJ) damping function.[13] Projector augmented wave (PAW) method[14] with a plane-wave basis set is employed to describe the interactions between ionic cores and valence electrons. For the plane-wave expansion to the electronic eigenfunctions, a cutoff energy of 500 eV is employed. To integrate the Brillouin zone, 6×6×1 Monkhorst-Pack (MP) mesh method[15] is used for k-point, determining the total energies and optimized geometries of the system. A catalytic surface is modeled with a supercell of slab. A vacuum space of 32 Å is added to minimize and separate the interactions between the periodic image of the surface and organic molecules (in z-direction).

The standard computational hydrogen electrode (CHE) method[16] describing the standard hydrogen electrode was implemented with an applied voltage (U), whereby electrons and protons at equilibrium at U = 0 V vs. CHE, describes the following:

$$\mathrm{H^+} + \mathrm{e^-} \rightarrow \mathrm{H_2}(g) \tag{1}$$

$$\frac{1}{2}\mu_{\mathrm{H^+}} - eU \rightarrow \mu_{\mathrm{H^+}} + \mu_{\mathrm{e^-}} \tag{2}$$

For instance, the change of free-energy, ΔG, of the proton-coupled electron (i.e., hydrogen) transfer for the reaction route of $OCCO^*$ to $HOCCO^*$ using Equation 2 can be defined and calculated via:

$$\Delta G = \mu_{HOCCO^*} - \mu_{OCCO^*} - \frac{1}{2}\mu_{\mathrm{H^+}} + eU \tag{3}$$

Herein, the change of free-energy, ΔG, can be calculated from:

$$\Delta G = \Delta E - T\Delta S + \Delta ZPE - eU \tag{4}$$

where ΔE is the change in total energy at T = 0 K computed from DFT calculations; ΔS is the change in entropy with T = 298.15 K; ΔZPE is the change in zero-point energy whereby vibrational contributions from frequency calculations are computed from DFT calculations.

We apply the VASPsol package[17], whereby the dielectric constant of water is set to 78.2 with a relative permittivity to describe the solvent medium, with which the vacuum space is described. Cu(111) with the structure of $8 \times 8 \times 4$ with the periodicity is used for the slab, while the 1-n-alkyl-3-methylimidazolium molecule is introduced on the surface of the slab after the Cu(111) structure is independently and geometrically optimized. Here, we note that due to the high computational cost of DFT calculations, we truncated the polymer backbone units and replaced the 1-n-alkyl-3-(4-vinylbenzyl)imidazolium groups of the styrene-ionomer units with 1-n-alkyl-3-methylimidazolium moieties with various alkyl chain lengths. For each system (slab), the surface contains one molecule of a 1-n-alkyl-3-methylimidazolium moiety with variations in their alkyl chain lengths (R), where R = C_nH_{2n+1} with n = 1, 4, 10, 16. The influence of conformational truncation on the energetics of the polymer backbone or benzyl group of styrene-ionomer units is beyond the scope of this study and, thus, is not investigated in this work. The adsorption energies are calculated with a 4-layer slab, whereby the bottom (top) two layers are fixed (relaxed). The relevant convergence tests are computed with respect to the thickness of the slab. All structures are geometrically optimized until the forces are < 0.05 eV Å^{-1}. To dissociate and minimize a fictitious interaction between the periodic images, a dipole correction is applied. The transition state energies and structures are determined through the climbing image nudged elastic band (CI-NEB) method[18], while the climbing image forces are converged with < 0.05 eV Å^{-1}.

3. Supporting Figures and Tables

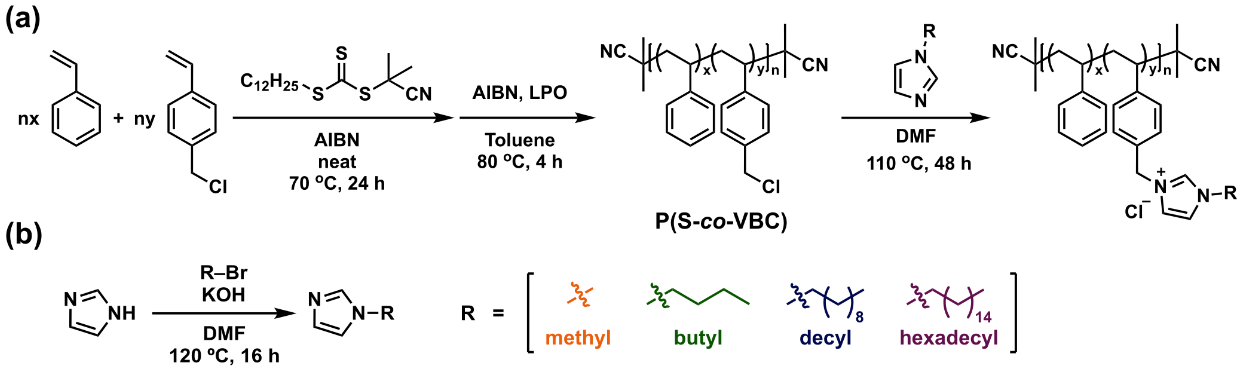

Figure S1. Synthetic schemes for (a) a series of 1-*n*-alkylimidazolium ionomers and (b) 1-*n*-alkylimidazoles with different alkyl side-chain lengths. The as-synthesized 1-*n*-alkylimidazoles were used to functionalize the P(S-*co*-VBC) to form the resulting series of 1-*n*-alkylimidazolium ionomers.

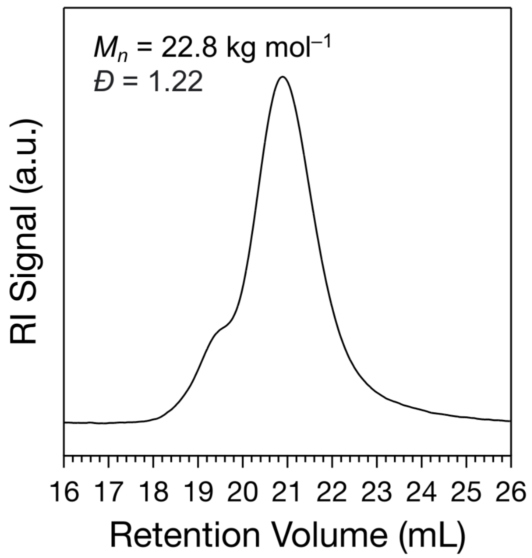

Figure S2. A GPC trace of the P(S-*co*-VBC) statistical copolymer. The molecular weight (M_n) and dispersity ($Đ$) of the polymer were determined to be 22.8 kg mol^{-1} and 1.22, respectively.

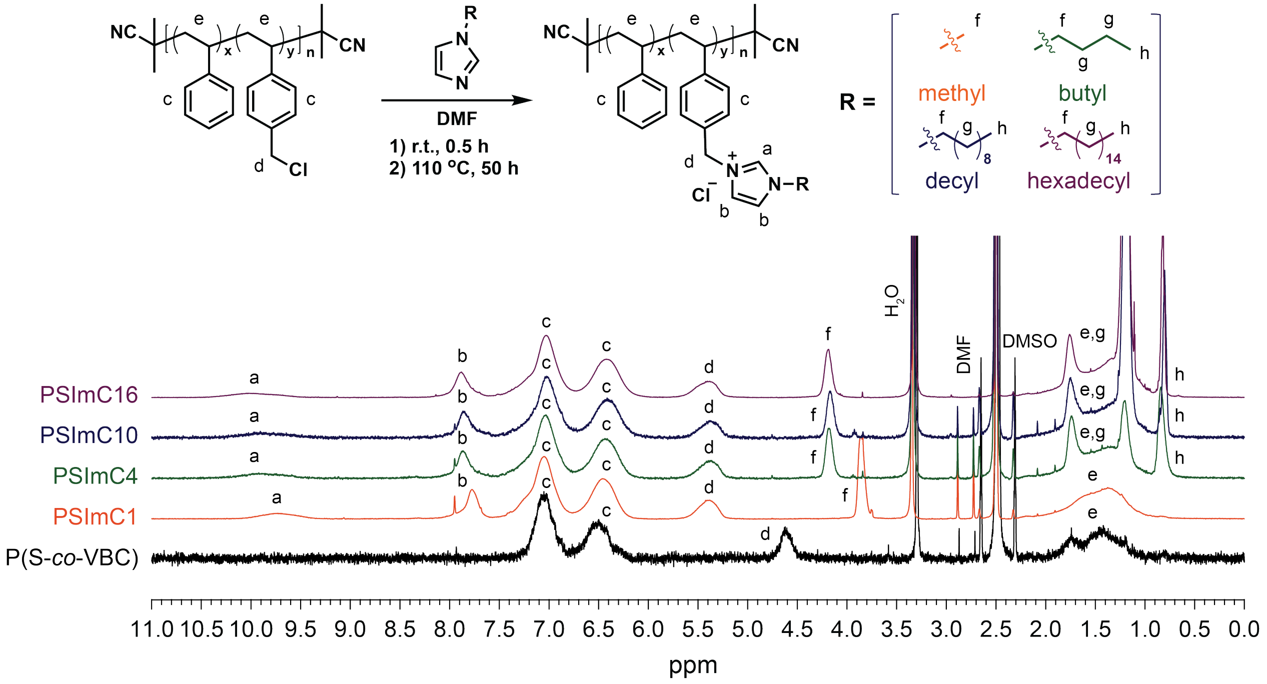

Figure S3. ^{1}H NMR spectra for the parent copolymer, P(S-*co*-VBC), and the functionalized 1-*n*-alkylimidazolium ionomers in DMSO−d_6: P(S-*co*-VBC) (black), PSImC1 (orange), PSImC4 (olive), PSImC10 (navy), and PSImC16 (purple). The mole fractions of the styrene and VBC components in the P(S-*co*-VBC) were confirmed to be 64.6 mol% and 35.4 mol%, respectively. All functionalization reactions have reached 100% conversion, as evidenced by the downfield shift of each benzylic methylene peak from 4.6 ppm to 5.4 ppm and the emergence of characteristic peaks that are attributed to the 1-*n*-alkylimidazolium groups.

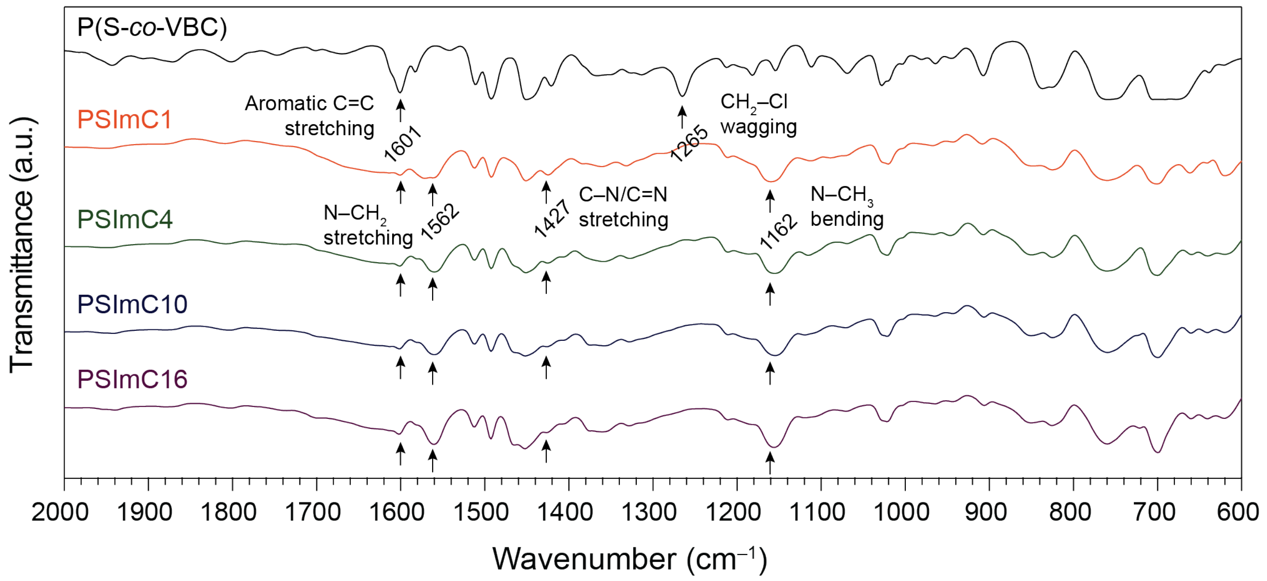

Figure S4. Infrared spectra of P(S-*co*-VBC) and functionalized 1-*n*-alkylimidazolium ionomers: P(S-*co*-VBC) (black), PSImC1 (orange), PSImC4 (olive), PSImC10 (navy), and PSImC16 (purple). Key absorption bands are observed at 1601 cm^{-1} (aromatic C=C stretching), 1562 cm^{-1} (N–CH_2 stretching), 1427 cm^{-1} (C–N/C=N stretching), 1265 cm^{-1} (CH_2–Cl wagging), and 1162 cm^{-1} (N–CH_3 bending). These peaks confirm the presence of aromatic rings, amine groups, and the absence of chloromethyl groups in the ionomers.

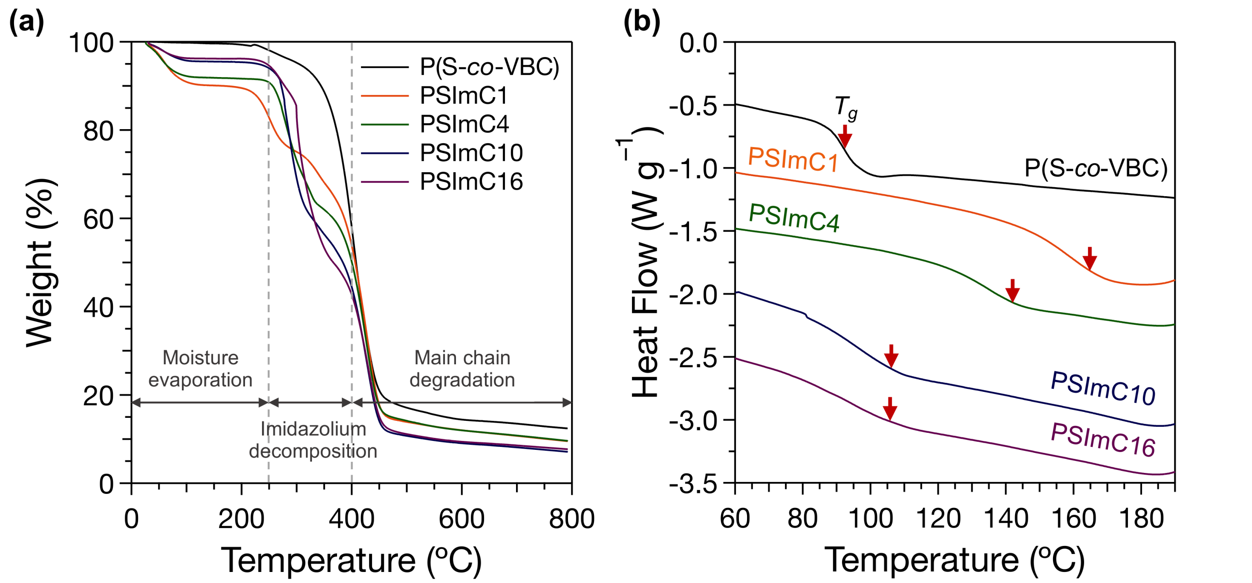

Figure S5. (a) Thermogravimetric analysis (TGA) and (b) differential scanning calorimetry (DSC) of the P(S-*co*-VBC) and 1-*n*-alkylimidazolium ionomers. The thermal stability of these ionomers was confirmed within the relevant temperature range for subsequent experimental procedures.

Table S1. RAFT copolymerization and characterization of the P(S-*co*-VBC).

Polymer	Initiator/CTA	Time (h)	Conv.[b] (%)	M_n[c] (g/mol)	M_w/M_n[c]	Composition[b] (%)
P(S-*co*-VBC)[a]	AIBN/CPDT	24	42	22,800	1.22	65% S, 35% VBC

[a] $[S]_0:[VBC]_0:[I]_0:[CTA]_0$ = 531:227:0.1:1. The RAFT polymerization was carried out at 70 ℃ without a solvent; [b] The polymerization conversion and composition of the polymer were determined using ^{1}H NMR analysis; [c] Molecular weight and dispersity were determined by GPC based on calibration with PS standards.

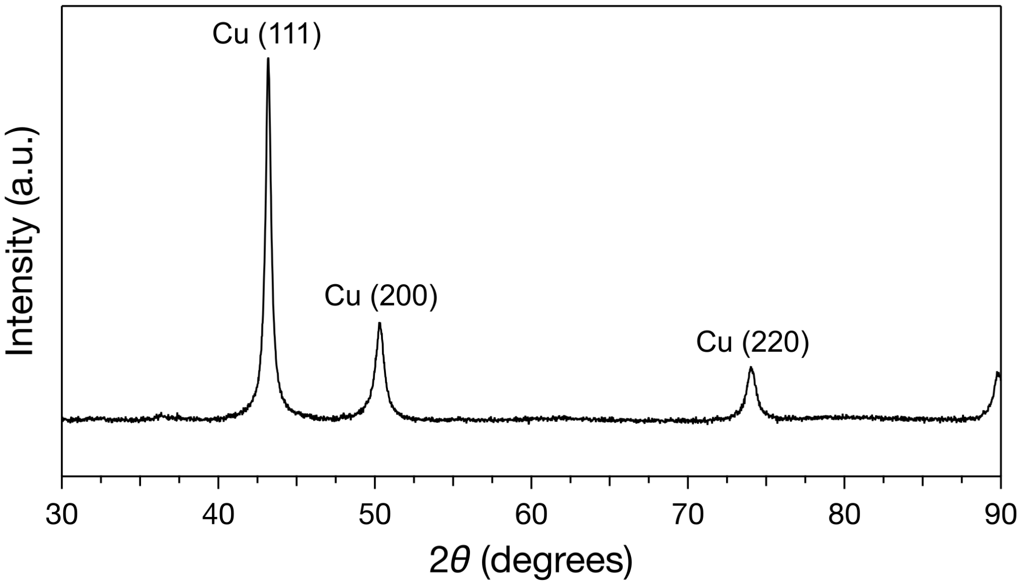

Figure S6. X-ray diffraction (XRD) patterns of commercial Cu NPs used in this study.

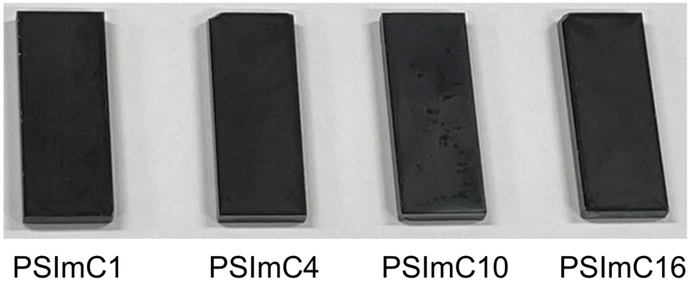

Figure S7. A photograph of the glassy carbon electrodes coated with the Cu NPs and 1-*n*-alkylimidazolium ionomers.

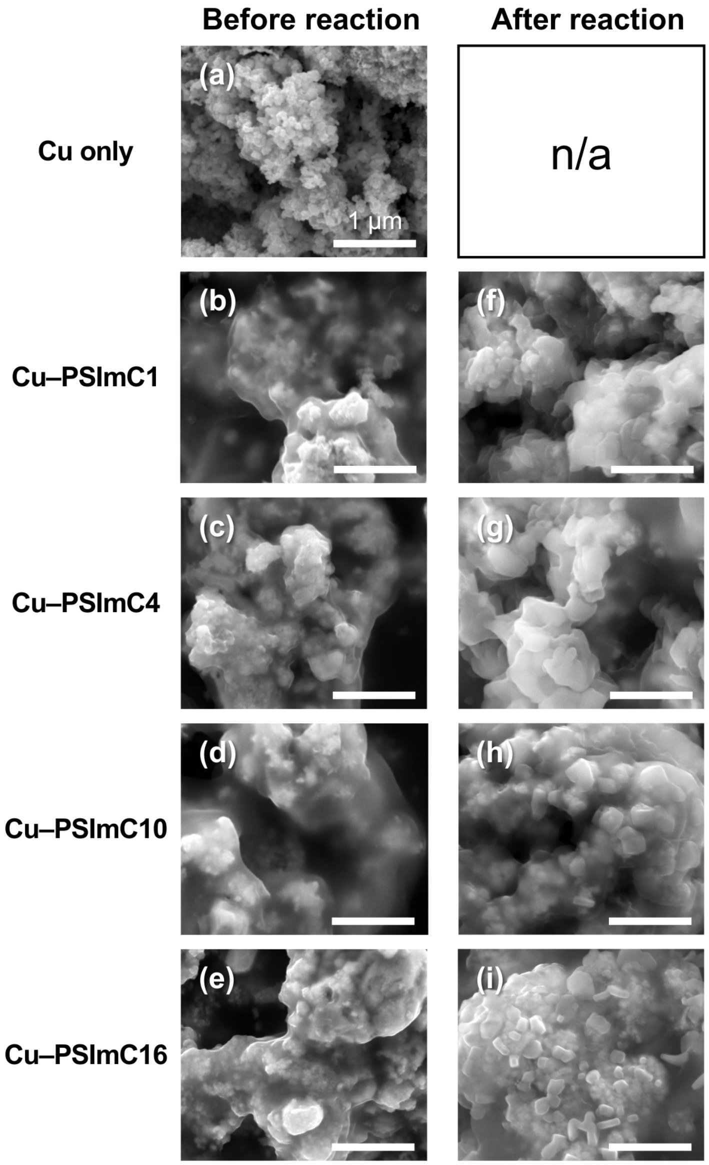

Figure S8. SEM images of electrodes (a–e) before and (f–i) after electrochemical CO_2 reduction (scale bar = 1 μm): (a) Cu NPs without ionomers and (b–i) Cu NPs coated with each 1-*n*-alkylimidazolium ionomer.

(a)

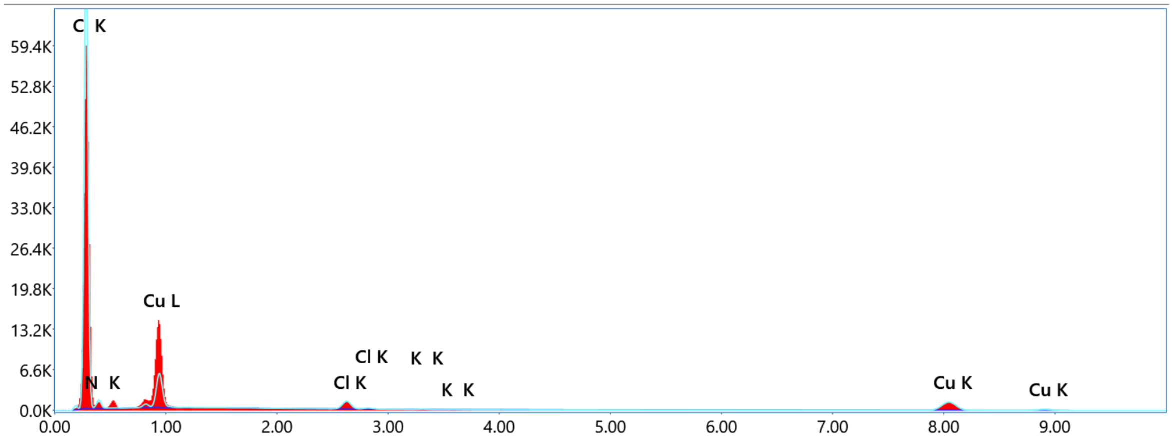

(b)

Element	Weight %	MDL	Atomic %	Error %	Net Int.	R	A	F
**C K**	84.7	0.07	93.1	8.6	2012.6	0.9298	0.2796	1.0000
**N K**	5.0	0.24	4.7	14.5	36.6	0.9349	0.0456	1.0000
**Cl K**	0.6	0.02	0.2	4.2	71.3	0.9655	0.9327	1.0193
**K K**	0.0	0.03	0.0	57.8	2.8	0.9700	0.9648	1.0385
**Cu K**	9.7	0.16	2.0	4.5	129.4	0.9896	0.9966	1.0942

Figure S9. (a) EDS analysis and (b) eZAF quantitative results of the as-prepared Cu–PSImC10 electrode.

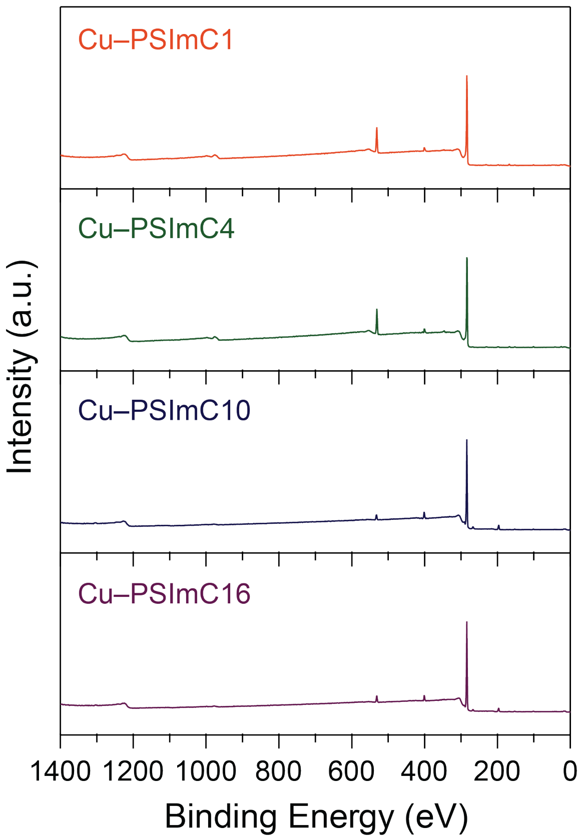

Figure S10. XPS survey spectra of the as-prepared electrodes coated with Cu NPs and different 1-*n*-alkylimidazolium ionomers.

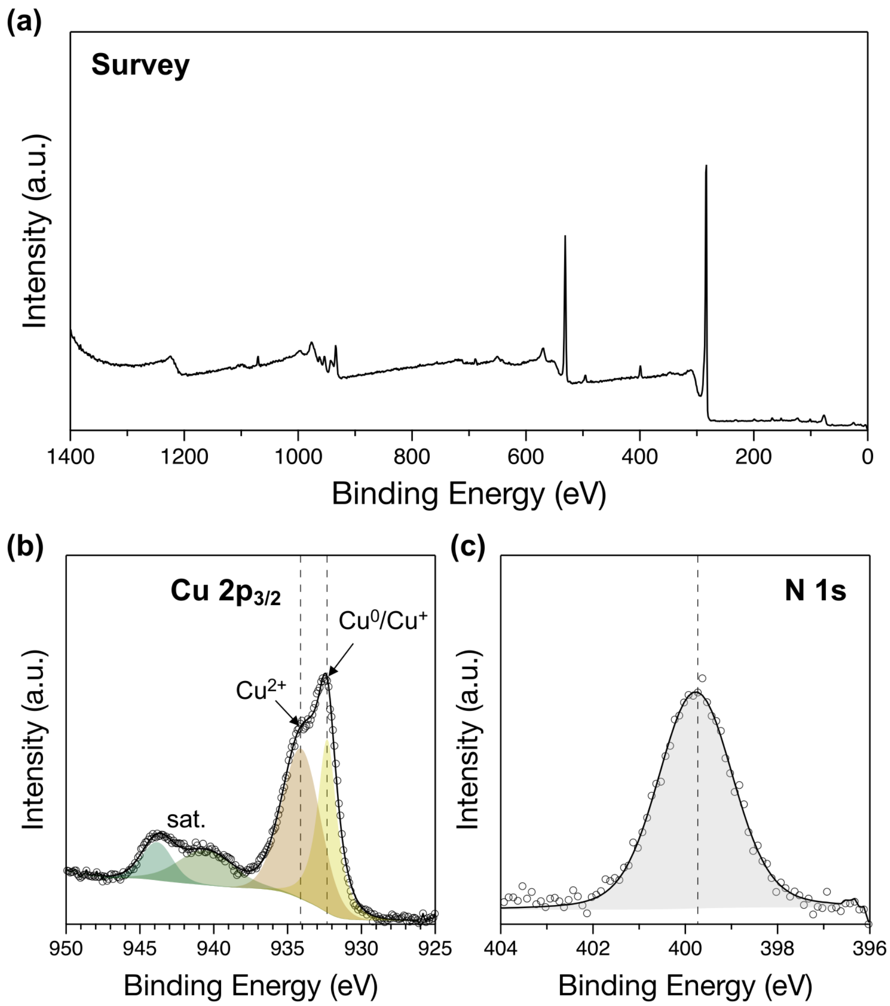

Figure S11. XPS spectra of the Cu NPs without ionomers (control sample) on a glassy carbon substrate: (a) survey, (b) Cu $2p_{3/2}$, and (c) N 1s.

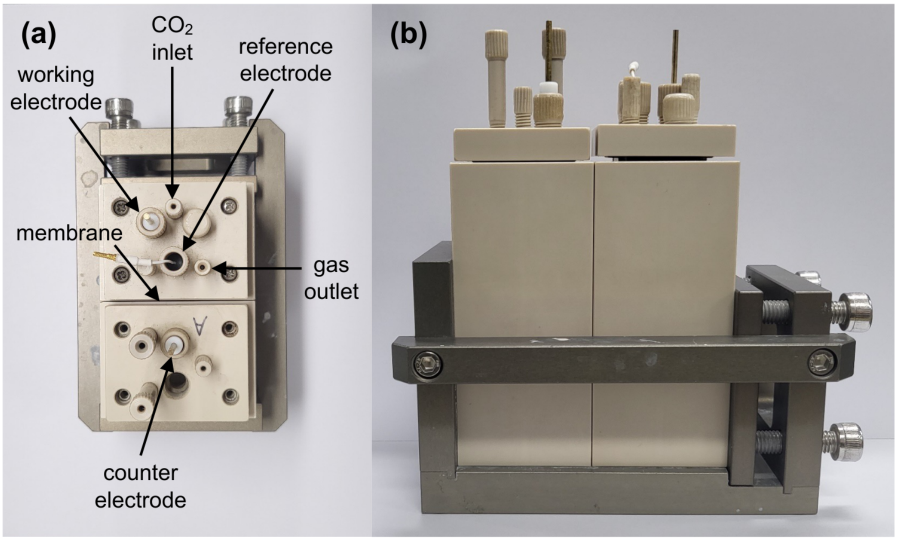

Figure S12. The two-compartment electrochemical cell was used in this study.

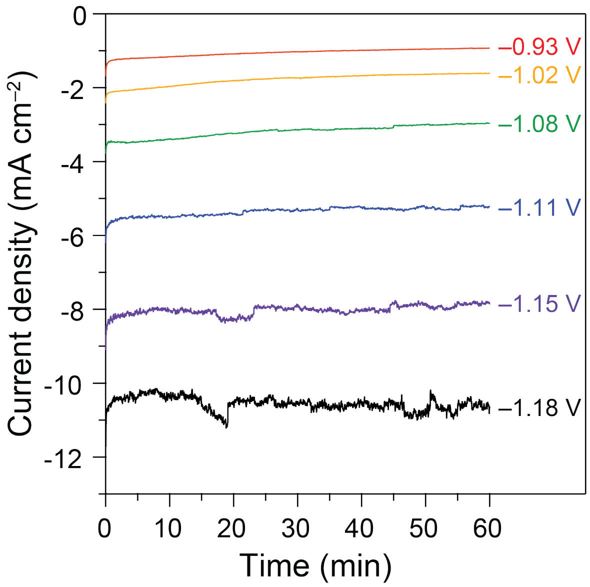

Figure S13. A representative total current density profile with time at various applied potentials on the Cu–PSImC10.

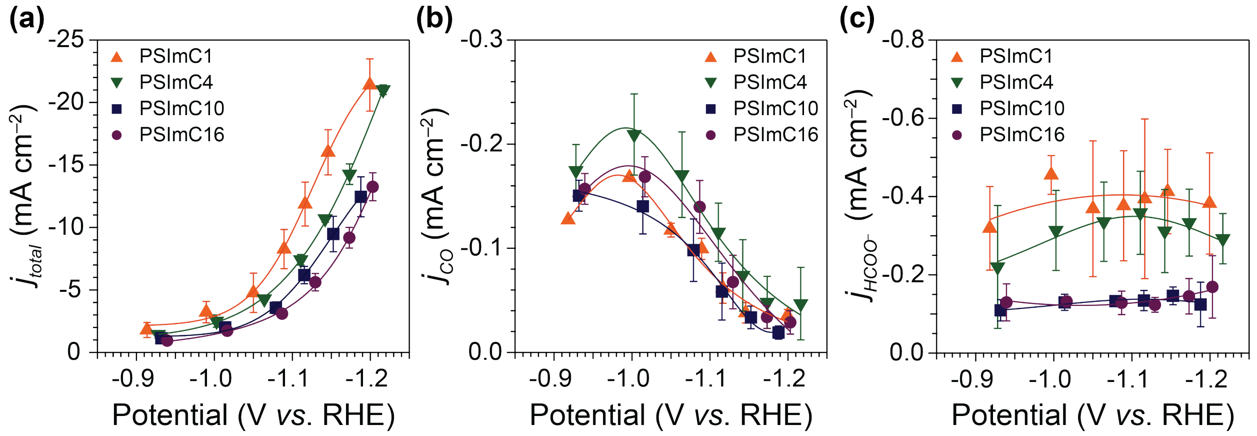

Figure S14. Catalytic activity and selectivity of the electrodes coated with the Cu NPs and 1-*n*-alkylimidazolium ionomers. (a) Total current densities during electrochemical CO_2 reduction at different applied potentials in a CO_2-saturated 0.1 M $KHCO_3$. (b,c) Partial current density profiles for (b) CO and (c) $HCOO^-$ production.

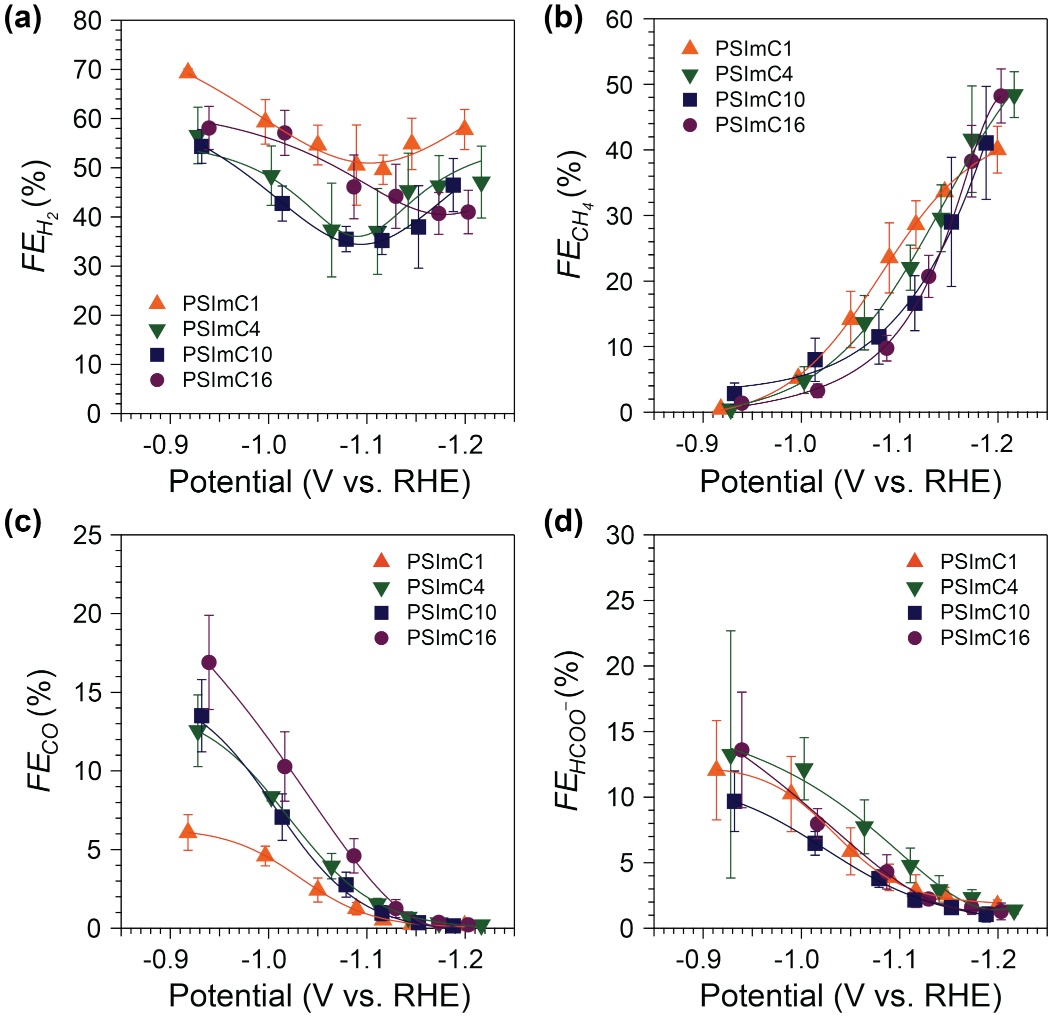

Figure S15. Faradaic efficiencies for (a) H_2, (b) CH_4, (c) CO, and (d) $HCOO^-$ at different applied potentials in a CO_2-saturated 0.1 M $KHCO_3$.

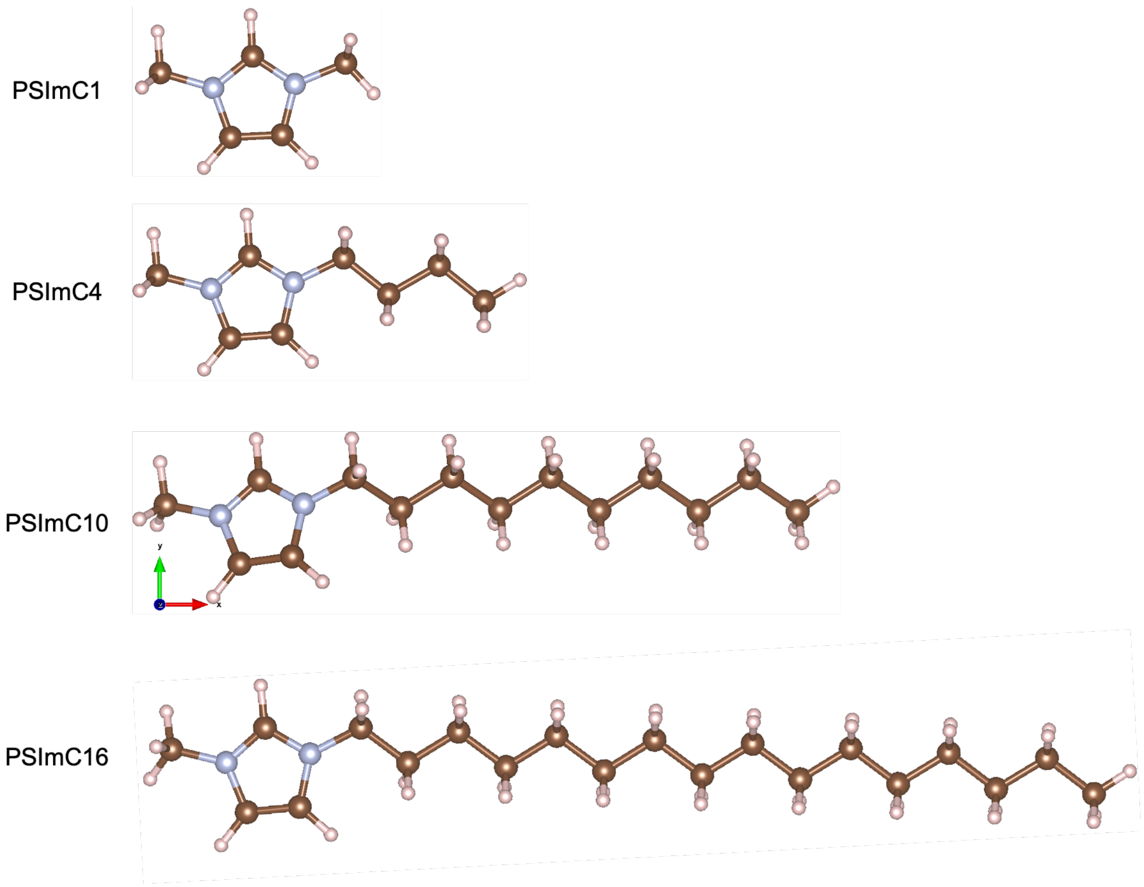

Figure S16. The geometrically optimized molecular structures of 1-*n*-alkyl-3-methylimidazolium moieties with various alkyl chain lengths (R), where $R = C_nH_{2n+1}$ with n = 1, 4, 10, 16 with PSImC1, PSImC4, PSImC10, PSImC16, respectively. As aforementioned, due to computational cost, we truncated the polymeric backbone and replaced the 1-*n*-alkyl-3-(4-vinylbenzyl)imidazolium groups of the styrene-ionomer units with 1-*n*-alkyl-3-methylimidazolium moieties. The geometry of each molecule is independently optimized prior to slab calculations.

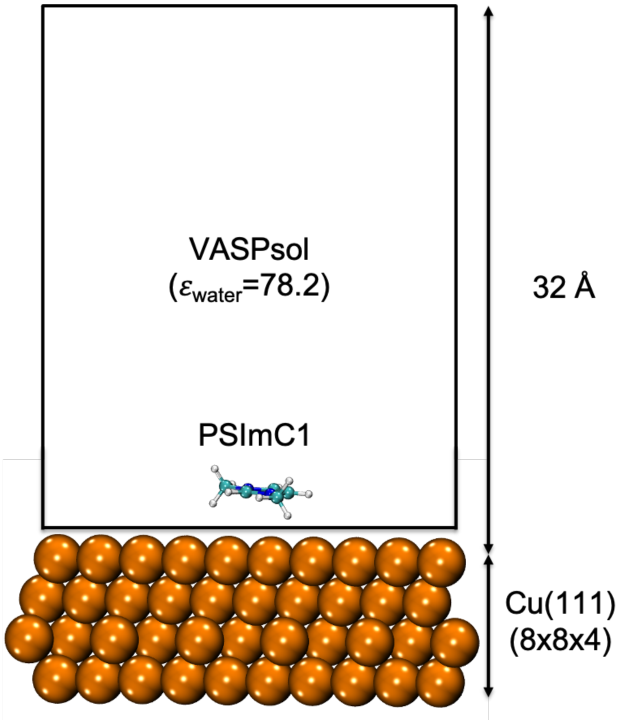

Figure S17. The system setup for DFT calculations with 8×8×4 of Cu(111) slab with one molecule of a 1-*n*-alkyl-3-methylimidazolium moiety with various alkyl chain lengths (R), where $R = C_nH_{2n+1}$ with n = 1 (that is, PSImC1, for example) with sufficient vacuum space representing implicit solvent water medium using VASPsol[17] with a dielectric constant, ε.

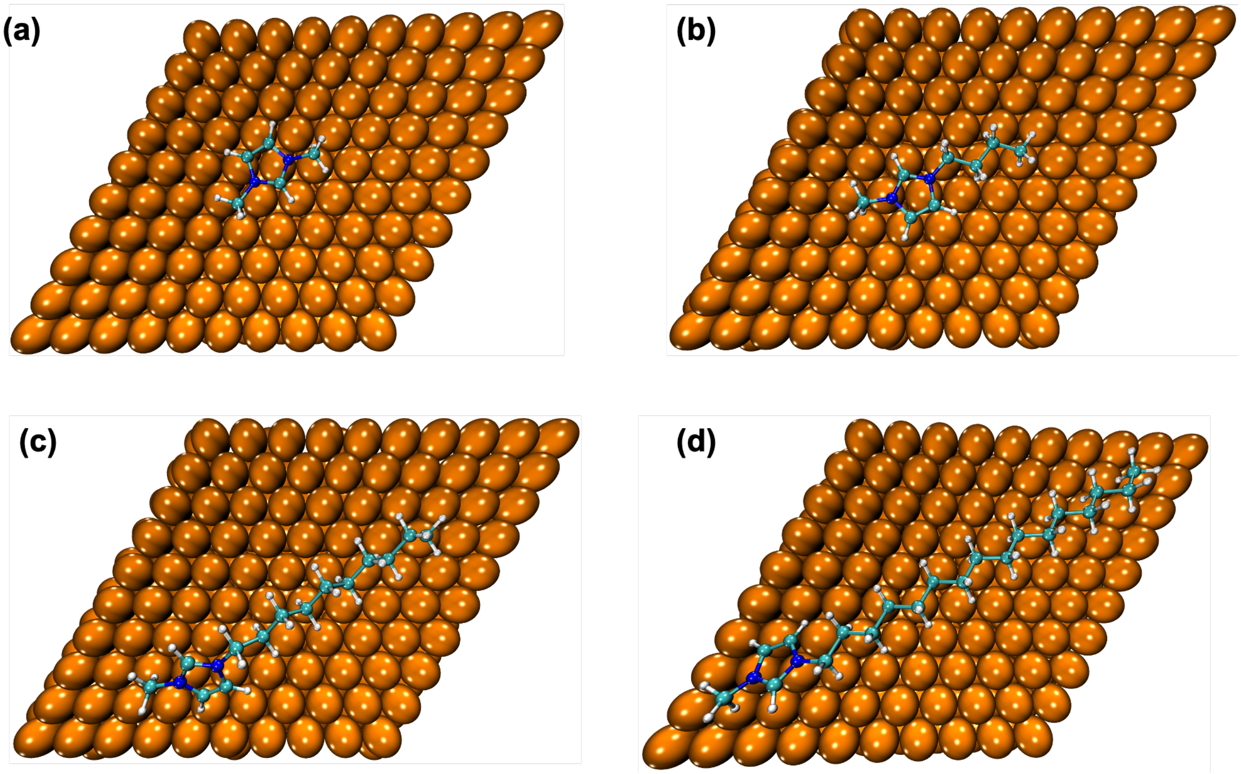

Figure S18. The system setup for DFT calculations with 8×8×4 of Cu(111) slab with one molecule of a 1-*n*-alkyl-3-methylimidazolium moiety of (a) PSImC1, (b) PSImC4, (c) PSImC10, and (d) PSImC16, in a bird's eye view.

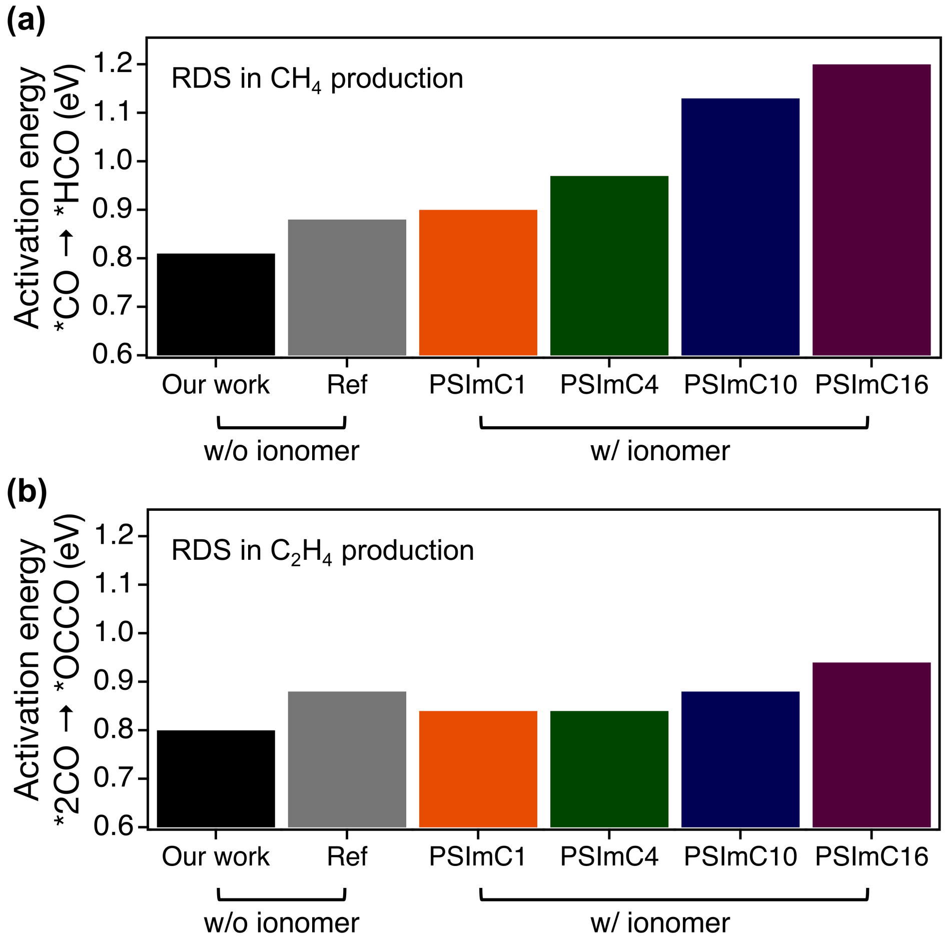

Figure S19. Calculated activation energy (in eV) from DFT calculations for the rate-determining steps (RDS) of (a) CH_4 and (b) C_2H_4 productions, with imidazolium moieties with various alkyl chain lengths (i.e., PSImC1, PSImC4, PSImC10, and PSImC16) and without an imidazolium moiety (our work), compared to the reference values.[19,20]

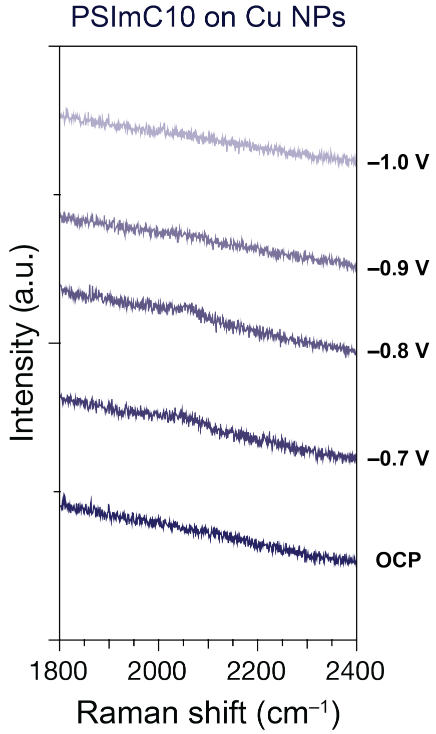

Figure S20. Representative in-situ Raman spectra of commercial metallic Cu NPs with the PSImC10 ionomer at indicated potentials (vs. RHE) in a CO_2-saturated 0.1 M $KHCO_3$ electrolyte. The spectra in the CO region (Raman shift between 1800–2400 cm^{-1}) were collected from the OCP to –1.0 V vs. RHE in the cathodic potential steps. The spectra from the metallic Cu NPs show no prominent features, except for a very small peak around 2060 cm^{-1} at –0.8 V. These Cu NPs were found unsuitable for analyzing CO_2RR intermediates due to the lack of a significant SERS effect.[4]

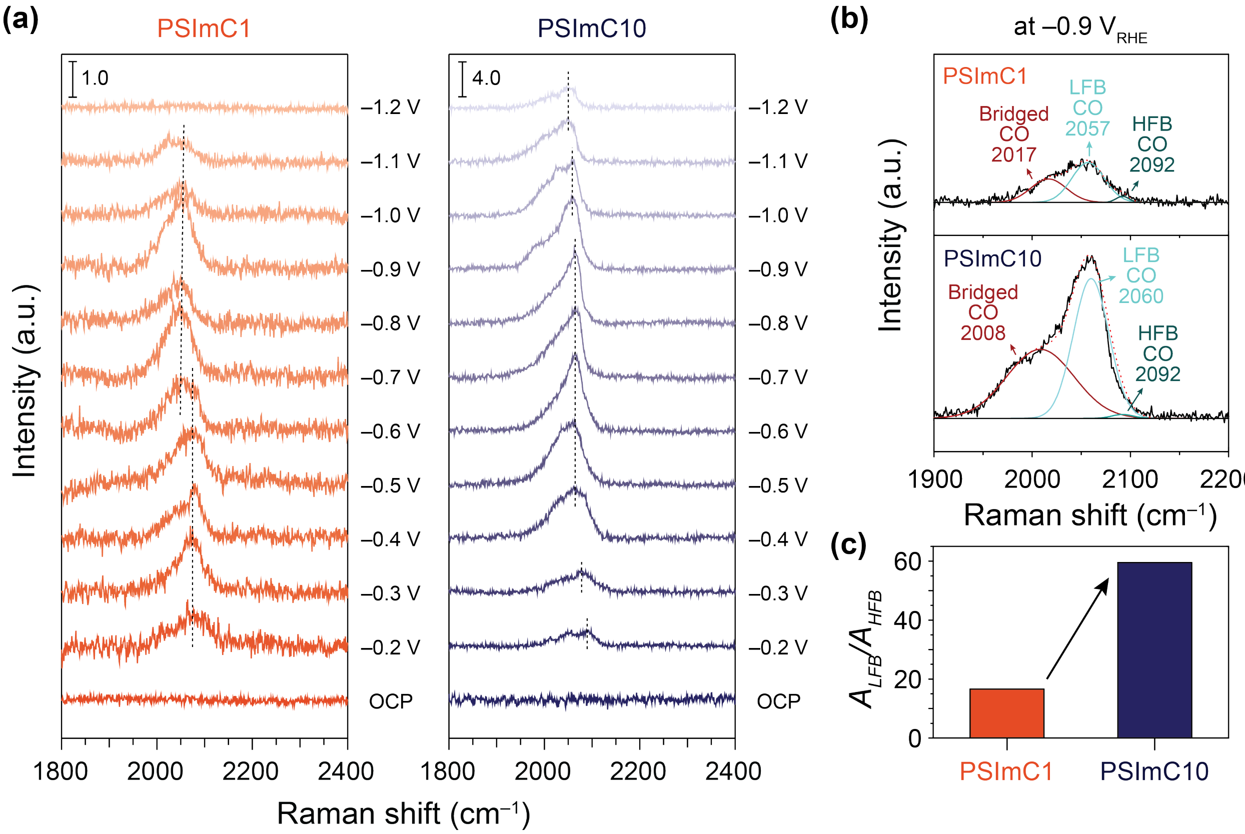

Figure S21. (a) In-situ Raman spectra of OD–Cu at indicated potentials (vs. RHE) in a CO_2-saturated 0.1 M $KHCO_3$ electrolyte with different ionomers: PSImC1 and PSImC10. The spectra in the CO region (Raman shift between 1800–2400 cm^{-1}) were collected from the OCP to –1.2 V in the cathodic potential steps. The CO peaks for both samples increase from the OCP to –0.9 V, and then fade from –1.0 V to –1.2 V. This behavior is likely due to the emergence of gas bubbles at the electrode surface across the higher overpotential region. (b) The spectrum at –0.9 V for each sample was thus deconvoluted into three peaks: bridged CO, low-frequency band (LFB) CO, and high-frequency band (HFB) CO.[6] (c) Comparison of the integrated area ratio between the LFB and HFB peaks (A_{LFB}/A_{HFB}) for both samples. The higher A_{LFB}/A_{HFB} ratio for PSImC10 suggests that C–C coupling and C_2H_4 production are more favorable on PSImC10 compared to PSImC1.[6]

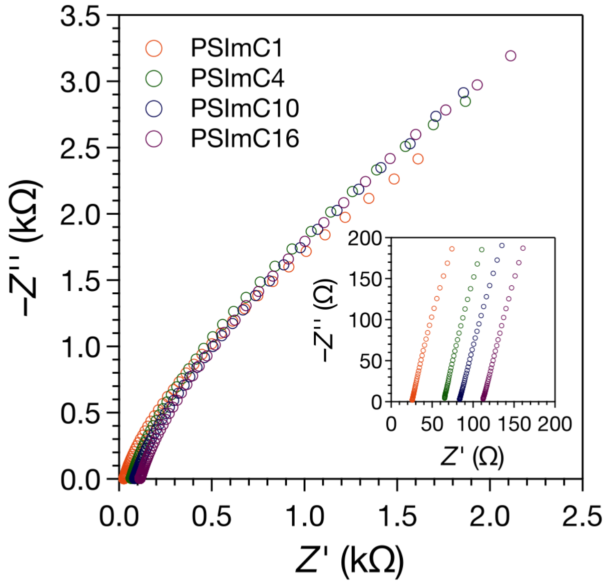

Figure S22. Electrochemical impedance spectroscopy (EIS) of 5 wt% solutions of the as-synthesized 1-*n*-alkylimidazolium ionomers in anhydrous DMSO (inset: magnified 0–200 Ω region). Solution resistance (R_s) was extracted from the spectra to determine the ionic conductivity of each sample.

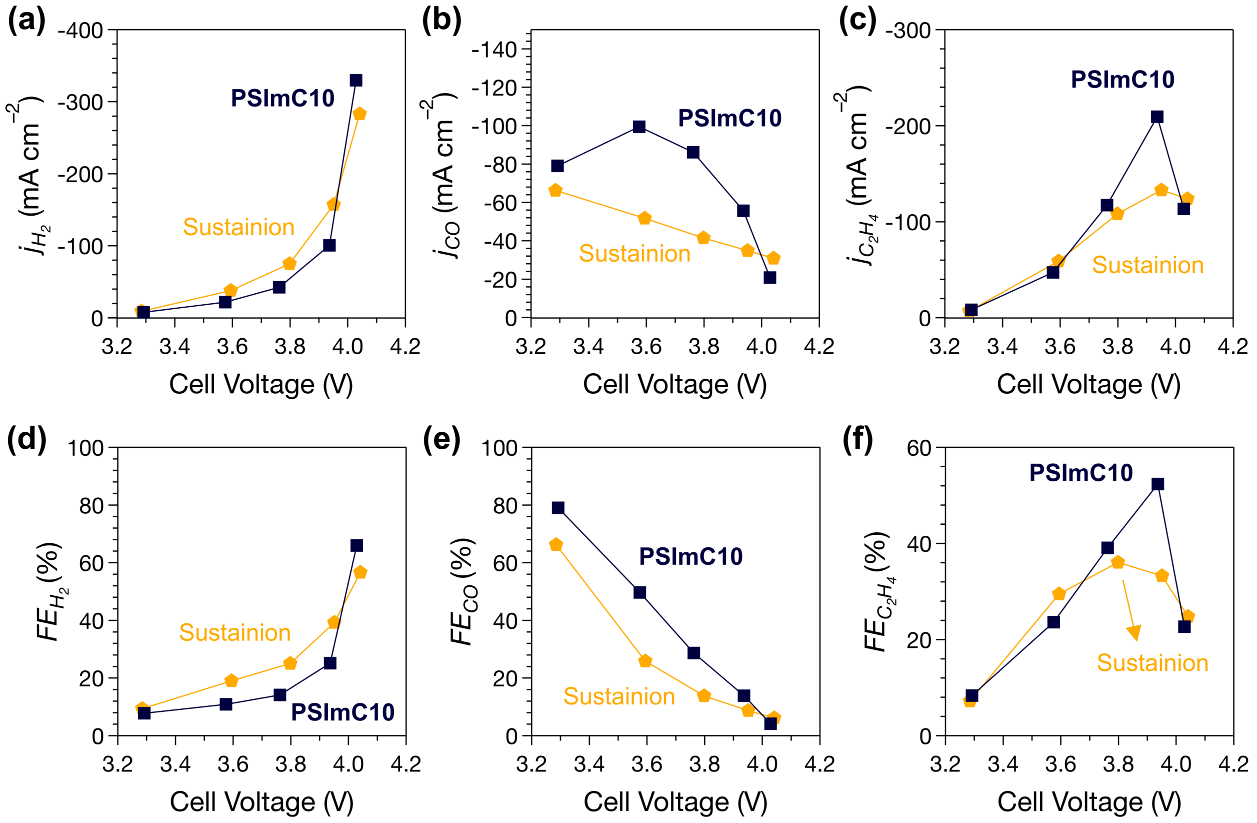

Figure S23. (a–c) Partial current densities (j) and (d–f) Faradaic efficiencies (FE) for major products (i.e., H_2, CO, and C_2H_4) during electrochemical CO_2 reduction in a membrane electrode assembly at various cell voltages. The PSImC10 sample is compared with the commercial Sustainion ionomer.

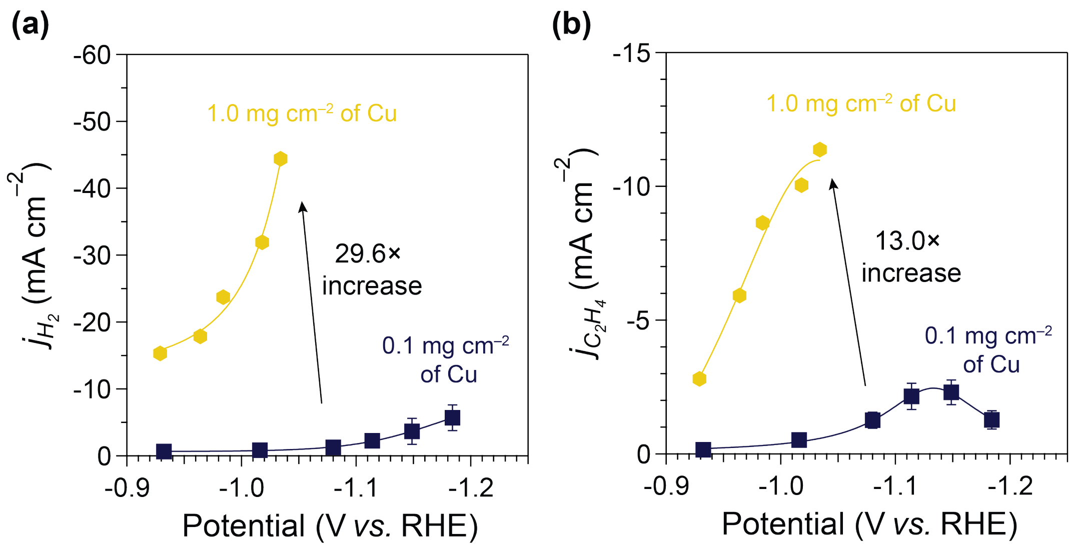

Figure S24. Comparison of catalytic activities for (a) HER and (b) C_2H_4 formation between different loading amounts of Cu NPs: 0.1 mg cm^{-2} and 1.0 mg cm^{-2}. The representative PSImC10 ionomer was used for this comparison. The electrochemical experiments were conducted across different applied potentials in a CO_2-saturated 0.1 M $KHCO_3$ aqueous electrolyte.

4. References

(S1) M. Chen, G. Moad, E. Rizzardo, *J. Polym. Sci. A Polym. Chem.* **2009**, *47*, 23 6704.

(S2) J.-Y. Cheng, Y.-H. Chu, *Tetrahedron Lett.* **2006**, *47*, 10 1575.

(S3) J. H. Koh, D. H. Won, T. Eom, N.-K. Kim, K. D. Jung, H. Kim, Y. J. Hwang, B. K. Min, *ACS Catal.* **2017**, *7*, 8 5071.

(S4) Y. Zhao, X. Chang, A. S. Malkani, X. Yang, L. Thompson, F. Jiao, B. Xu, *J. Am. Chem. Soc.* **2020**, *142*, 21 9735.

(S5) C. W. Li, J. Ciston, M. W. Kanan, *Nature* **2014**, *508*, 7497 504.

(S6) H. An, L. Wu, L. D. B. Mandemaker, S. Yang, J. de Ruiter, J. H. J. Wijten, J. C. L. Janssens, T. Hartman, W. van der Stam, B. M. Weckhuysen, *Angew. Chem. Int. Ed.* **2021**, *60*, 30 16576.

(S7) P. Hohenberg, W. Kohn, *Phys. Rev.* **1964**, *136* B864.

(S8) W. Kohn, L. J. Sham, *Phys. Rev.* **1965**, *140* A1133.

(S9) G. Kresse, J. Furthmüller, *Comput. Mater. Sci.* **1996**, *6*, 1 15.

(S10) G. Kresse, J. Furthmüller, *Phys. Rev. B* **1996**, *54* 11169.

(S11) J. P. Perdew, K. Burke, M. Ernzerhof, *Phys. Rev. Lett.* **1996**, *77* 3865.

(S12) S. Grimme, J. Antony, S. Ehrlich, H. Krieg, *J. Chem. Phys.* **2010**, *132*, 15 154104.

(S13) A. D. Becke, E. R. Johnson, *J. Chem. Phys.* **2005**, *123*, 15 154101.

(S14) G. Kresse, D. Joubert, *Phys. Rev. B* **1999**, *59* 1758.

(S15) H. J. Monkhorst, J. D. Pack, *Phys. Rev. B* **1976**, *13* 5188.

(S16) J. K. Nørskov, J. Rossmeisl, A. Logadottir, L. Lindqvist, J. R. Kitchin, T. Bligaard, H. Jónsson, *J. Phys. Chem. B* **2004**, *108*, 46 17886.

(S17) K. Mathew, V. S. C. Kolluru, S. Mula, S. N. Steinmann, R. G. Hennig, *J. Chem. Phys.* **2019**, *151*, 23 234101.

(S18) G. Henkelman, B. P. Uberuaga, H. Jónsson, *J. Chem. Phys.* **2000**, *113*, 22 9901.

(S19) Q. Chang, J. H. Lee, Y. Liu, Z. Xie, S. Hwang, N. S. Marinkovic, A.-H. A. Park, S. Kattel, J. G. Chen, *JACS Au* **2021**, *2*, 1 214.

(S20) Y. Xie, P. Ou, X. Wang, Z. Xu, Y. C. Li, Z. Wang, J. E. Huang, J. Wicks, C. McCallum, N. Wang, Y. Wang, T. Chen, B. T. W. Lo, D. Sinton, J. C. Yu, Y. Wang, E. H. Sargent, *Nat. Catal.* **2022**, *5*, 6 564.
